# Bacterial transfer during sexual intercourse as a tool for forensic detection

**DOI:** 10.1016/j.isci.2025.111861

**Published:** 2025-02-12

**Authors:** Ruby Dixon, Siobhon Egan, Matthew Payne, Christopher Mullally, Brendan Chapman

**Affiliations:** 1 Medical, Molecular and Forensic Sciences, Murdoch University, 90 South Street, Murdoch, WA 6150, Australia; 2 Australian National Phenome Centre and Computational and Systems Medicine, Health Futures Institute, Harry Perkins Institute of Medical Research, Murdoch, WA 6150, Australia; 3 Division of Obstetrics and Gynaecology, University of Western Australia, Crawley, WA 6009, Australia; 4 Wesfarmers Centre for Vaccines and Infectious Diseases, Telethon Kids Institute, Nedlands, WA 6009, Australia

**Keywords:** Biological sciences, Microbiome

## Abstract

In forensic science, detecting transfers of physical and biological material is critical for establishing evidence of criminal involvement. Unique bacterial signatures from the reproductive system transfer during unprotected penetrative intercourse offer a novel tool for criminal investigation. Here, we demonstrate this transfer using full-length 16S rRNA gene sequencing and discuss the impact of barrier contraceptives. These microbial signatures can potentially aid in sexual assault casework for perpetrator identification when human male DNA is absent.

## Highlights

• Transfer of unique and non-unique bacterial taxa during intercourse was observed
• *Lactobacillus* spp. contributed to the most female to male sexome transfer
• Despite the use of a condom during intercourse, transfer of the sexome was still observed
• Condom/lube use, oral intercourse, circumcision/pubic hair: no impact on microbial diversity

## Graphical abstract

**Figure:**
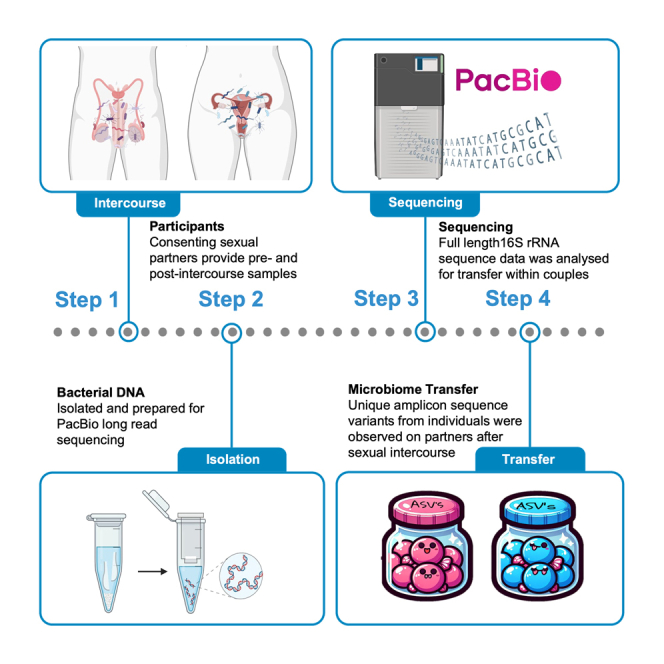

## Introduction

Sexual assault remains a pressing public health and safety concern that affects an estimated one in three women globally.^1^ Sexual assault is a broad term used to describe any form of non-consensual sexual contact ranging from assault by physical force or incapacitation to contact without ongoing consent.^2^ While this kind of assault occurs regardless of biological sex, gender identity, or sexual orientation, women face a higher risk. Prevalence rates of global male sexual assault are near impossible to provide given the underreported nature of these crimes.^3^ In Australia, one in six women aged 15 years and above have experienced sexual assault, whereas the figure for men stands lower at 1 in 25.^4^ The Crime Survey for England and Wales reported one in four women and one in 18 men had been subject to some kind of sexual violence since the age of 16 years.^5^ Similarly, in the United States one in four women and one in 26 men have experienced an attempted or completed rape in their lifetime.^6^ While victims are most often women, the majority of sexual offenders in Australia are recorded as male.^4^

The investigation of sexual assault relies heavily on isolating perpetrator DNA from spermatozoa, typically retrieved through a forensic medical examination of the female victim. It is crucial to obtain biological specimens as soon as appropriate, as vaginal swabs taken after 24 h are less likely to contain sperm that can be isolated and analyzed.^7^^,^^8^^,^^9^ Human DNA evidence plays a critical role in sexual assault cases where the perpetrator is either unidentified by the victim or denies engaging in the contact. While obtaining an autosomal DNA profile for searching against large databases is crucial, it is not without limitations. Due to the environment in which biological specimens are sampled from, namely the vagina, mixed biological samples are unavoidable. Often there is an overabundance of female “self” DNA derived from vaginal epithelial cells which influences the ability to obtain a male autosomal short tandem repeat (STR) profile.^10^ To address this problem, the isolation of male DNA is targeted using a technique called differential extraction. While this method is widely adopted for DNA isolation in sexual assault cases worldwide it is imperfect. Despite many variations to the method, it often fails to adequately separate male and female cellular contributions, is labor-intensive and time-consuming, and may result in the degradation of the male DNA within the sample.^8^^,^^10^^,^^11^^,^^12^^,^^13^^,^^14^ An alternative method to counter issues with mixed DNA samples is by targeted STR profiling of the male-specific Y chromosome. Limitations still exist with this method due to the paternally inherited nature of the Y chromosome, along with smaller Y-STR databases compared to autosomal ones. Despite multiple options within the forensic toolkit, the retrieval of a male DNA profile remains challenging. This highlights the necessity for additional methods to assist in identifying perpetrators in sexual assault investigations.

The application of the human microbiome to forensic investigation is an active area of research.^15^^,^^16^^,^^17^^,^^18^^,^^19^ Previous studies have reported that, while vastly interconnected, human bacterial composition differs between body sites and among individuals.^20^^,^^21^^,^^22^ This creates an opportunity to assess whether individuals carry a unique microbial signature that can be detected and used forensically. The bacterial 16S ribosomal RNA (16S rRNA) gene is ∼1,500 bp in length and commonly targeted in microbiome studies. This is due to its presence in the core genome of bacteria and the nine conserved regions that provide universal primer binding opportunities.^23^ Short-read DNA sequencing approaches have dominated microbiome studies in recent years due to their high accuracy and affordability. However, identifying bacterial taxa at a species level using these techniques is challenging and, therefore, limits the forensic capability of this research.^24^ For the microbiome to be used forensically to identify unique microbial signatures in individuals, bacterial species, and subspecies resolution is vital.^25^^,^^26^^,^^27^ Pacific Biosciences (PacBio) Single-Molecule Real-Time (SMRT) sequencing, now commonly referred to as HiFi sequencing, allows for highly accurate sequencing of the full-length 16S rRNA gene with species-level resolution of many bacterial taxa. In fact, a recent study that employed the use of full-length 16S rRNA gene sequencing was able to resolve some species to the serovar level through the use of additional biostatistical tools.^25^

Few studies have explored the analysis of vaginal and penile microbiomes within a forensic context. Tridico et al.^28^ was the first to publish a comprehensive metagenomic analysis of the bacteria found on human hairs from the scalp and pubic region in assessment for use in forensic applications. Williams et al.^29^ investigated the bacterial communities of pubic hairs from six individuals (three female and three male). Interpersonal variation was observed with more prominent variation between sexes. A successive study by the same authors looked at the shared microbiome in coinhabiting and non-coinhabiting couples.^16^ This involved the collection of swabs and hairs from the pubic mound of individuals and couples. It found a significant correlation between the proportion of shared bacterial taxa and the average number of times the couple engaged in sexual intercourse during the week prior to sample collection. The study, however, was unable to identify direct microbial transfer that occurs during penetrative intercourse, nor were parameters set to imitate a one-off sexual encounter.^16^ A study by Neckovic et al.^17^ observed the direct (skin to skin) and indirect (skin to vector to skin) transfer of the skin microbiome between individuals. Transfer of the microbiome from the hands occurred irrespective of the mode of transfer (direct and indirect) and for all substrates used to facilitate indirect transfer. These studies show that transfer of the native microbiome can be observed and potentially used within a forensic context. A pilot study of the sexual microbiome, or ‘sexome’, utilizing short-read analysis of the 16S rRNA gene V3-4 region was recently conducted to investigate microbial transfer during intercourse.^18^ Parameters were set to simulate a once-off sexual encounter between monogamous partners. It found that couples’ sexomes clustered closer together *after* intercourse and not *before*, suggesting microbial communities became more homogeneous *after* sexual contact. Amplicon sequence variants (ASVs) that were unique to the individuals were detected with some subsequently detected in their sexual partner following intercourse. These findings provided a proof of concept that bacterial signatures transfer during sexual contact. True to the forensic principle that “every contact leaves a trace”,^30^ these bacterial traces can be detected from sampling following intercourse. A limitation of the Dixon et al. sexome study, however, was that it could not accurately identify bacterial taxa beyond genus level, a limitation of short read analyses. For the microbiome to be used forensically, a method must be developed to correctly identify species, sub-species and serovar levels of bacterial taxa. The present study was designed to investigate the application of long-read bacterial 16S rRNA gene sequencing in identifying microbial signatures that transfer during intercourse.

## Results and discussion

### Participant demographics

Couples were aged between 20 and 30 years of age and used a variety of different contraceptive methods such as the oral contraceptive, condoms, and the withdrawal method [Table 1]. Couples reported abstaining from intercourse for between two and 14 days prior to intercourse as part of the study.

**Table 1:** Summary of participant information

Couple	Participant Information	Contraception	Time Abstinent Prior to Intercourse
1	Male = unknown yrs	Contraceptive Pill	5 days
	Female = 22 years		
2	Male = 25 years	Contraceptive Pill	10 days
	Female = 25 years		
3	Male = 30 years	Condom (started without)	10 days
	Female = 28 years		
4	Male = 23 years	Condom	5 days
	Female = 26 years		
5	Male = 22 years	Contraceptive Pill & Condom	6 days
	Female = 22 years		
6	Male = 29 years	Vasectomy	Unknown
	Female = 27 years		
7	Male = 28 years	Condom	7 days
	Female = 25 years		
8	Male = 22 years	Contraceptive Pill	3 days
	Female = 28 years		
9	Male = 23 years	Contraceptive Pill	2 days
	Female = 22 years		
10	Male = 23 years	Contraceptive Pill	2 days
	Female = 22 years		
11	Male = 22 years	Contraceptive Pill	3 days
	Female = 22 years		
12	Male = unknown yrs	Contraceptive Pill	4 days
	Female = 22 years		

Questions where a participant failed to provide information have been marked as unknown. Further demographic information can be found in the Table S2.

### Bioinformatics

A total of 681,848 circular consensus sequences (CCS) were produced from 48 samples and 6 controls (one positive control, three extraction blank controls, and two negative template controls). Sequences were quality filtered and the DADA2 denoise algorithm was used to produce ASVs. A total of 2,621 ASVs were identified. After taxonomic assignment, a total of 447,758 bacterial sequences were identified (mean read count = 8,292 reads).

### Data quality control

The *decontam* package identified a total of 45 out of 2,621 ASVs as contaminants. It is well reported that DNA extraction processes impart low level microbial contamination.^31^^,^^32^ The *decontam* package corrects for this by identifying ASVs common to samples and controls. Once identified, these contaminants were removed from the data [Table S4]. The performance of the extraction protocol was evaluated using an artificially constructed community of known composition (mock community). The ZymoBIOMICS mock community yielded 45,338 CCS reads above the *minPasses* threshold of 3 and the *minPredictedAccuracy* threshold of 99.9%, and 27,993 reads after removing primers and filtering. In the Zymo mock community, 30 ASVs were detected, of which 15 were exact matches (100% identity, 100% coverage) to 16S rRNA genes from the reference strains provided by Zymo Research. The remaining 15 ASVs were exact matches to 16S rRNA genes of the expected species which is likely an example of sequence variation within 16S copies. The relative abundances of the ZymoBIOMICS mock community detected from our protocol were *Staphylococcus aureus* (20%), *Listeria monocytogenes* (18.1%), *Salmonella enterica* (17.3%), *Enterococcus faecalis* (13.9%), *Escherichia coli* (13.6%), *Bacillus subtilis* (9.7%), *Pseudomonas aeruginosa* (4.9%), and *Lactobacillus fermentum* (2.3%) [Figure S3].

### Alpha diversity analysis of the vagina and penile skin microbial community

**Figure 1:**
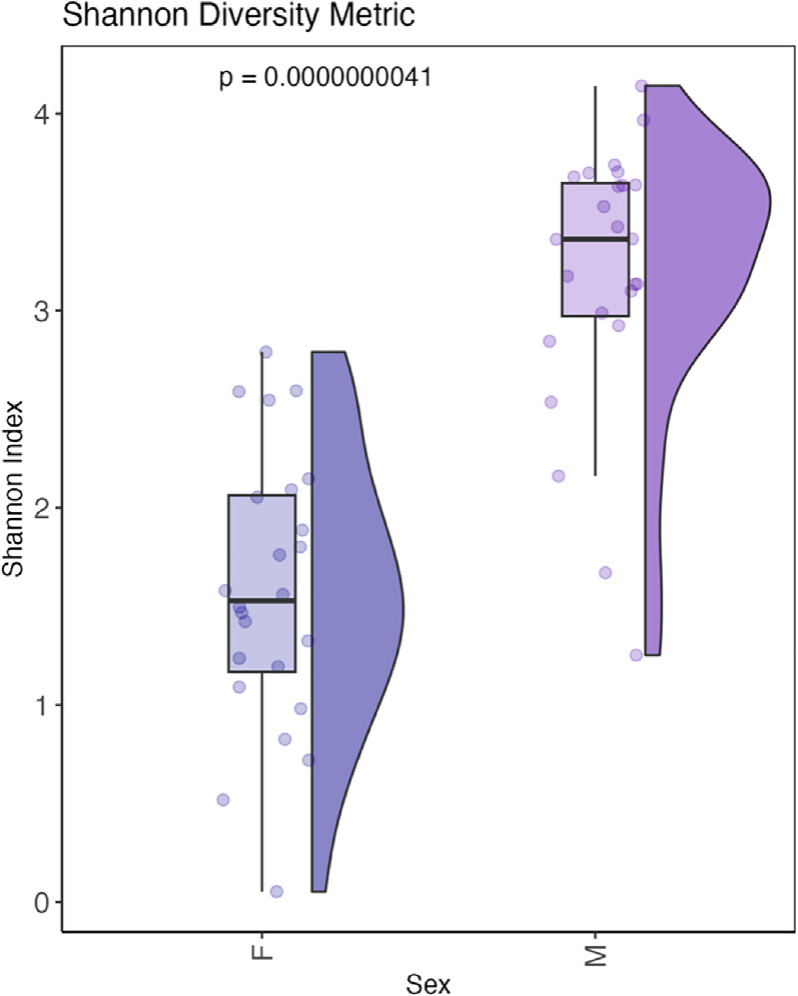
Alpha diversity plot of microbial communities grouped by sex displayed using Shannon diversity measures Statistical significance calculated using the Wilcoxon test (*p* = 0.0000000041).

Alpha diversity analysis identified significant variation between male and female samples (Wilcoxon pairwise test) [Figure 1].^33^ Overall male and female samples had an average of 6,661 and 8,038 bacterial sequences, respectively. While a higher number of sequences were retrieved from female vaginal samples, penile skin communities were significantly more diverse, having approximately two times more bacterial taxa (ASVs) in total (*n* = 1,059) than vaginal samples (*n* = 514). Male samples consistently showed the highest alpha diversity across all metrics used (*p* < 0.05) [Figure 1].

The vaginal bacterial microbiome has been more heavily investigated in contrast to the male penile microbiome. A meta-analysis of vaginal microbiome studies showed that the environment was relatively low in diversity in most women, with dominance by a few bacterial genera, typically *Lactobacillus*.^34^^,^^35^ In contrast, while fewer studies have been conducted on the penile microbiome they suggest a relatively higher diversity in bacterial taxa.^36^ This is reflected in the diversity of the skin microbiome^37^ and it is unsurprising that the skin of the penis more closely reflects bacterial diversity seen across skin than within the vagina. In addition, the most dominant bacteria residing on the penile skin are taxa that can exist in aerobic environments which include *Staphylococcus* and *Corynebacterium*.^38^ Variation in the penile skin of circumcised and uncircumcised males has been reported.^39^^,^^40^^,^^41^ This is due to the penile prepuce in uncircumcised males which forms an environment where potentially pathogenic anaerobic bacteria can proliferate. Taxa reported in this environment include *Prevotella*, *Porphyromonas*, *Anaerococcus*, *Streptococcus* and *Finegoldia*.^42^^,^^43^^,^^44^^,^^45^ Compared with the female vagina, which is an entirely anaerobic environment, the diversity is vastly different and consists of strict and facultatively anaerobic bacteria.^46^ The large difference in physical conditions of the two sexual organs allow for transfer of taxa not ordinarily seen on both. Vaginal fluid has been investigated as method of detecting sexual transfer in other forensic studies with similar justification.^47^^,^^48^^,^^49^

### Disruption to microbial diversity

**Figure 2:**
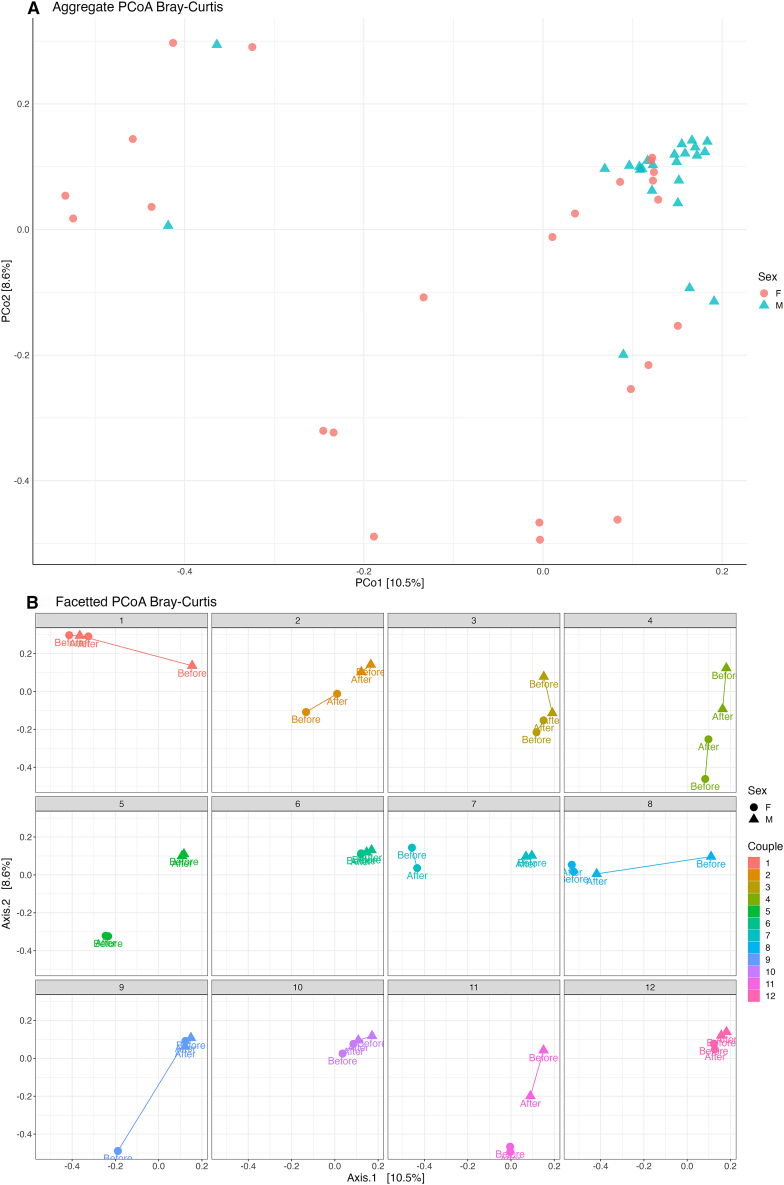
Principal coordinate analysis (PCoA) plots (A) PCoA based on the Bray-Curtis distance measure^51^ of 1,061 ASVs and 48 samples. Prior to the analysis, ASVs not present in more than 0.1% relative abundance in any sample were removed. No initial data transformation was applied. The relative contribution (eigenvalue) of each axis to the total inertia in the data is indicated in percent at the axis titles. Each point represents a sample, with colors indicating couple, shape indicating sex and a label to show which point corresponds to each sampling time (“*Before*” and “*After*” intercourse). (B) PCoA plot as described above displaying individual couples, to allow for a visual comparison of sample distributions across sex and sampling time within each couple. A line is used to connect each sample from the same sex to show the change in diversity between sampling times.

Beta-diversity measures shown by ordination analysis indicated a difference in the composition of bacterial taxa in male and female samples [Figure 2A].^50^ Principal coordinate analysis using the Bray-Curtis metric, which assesses dissimilarity based on overall ASV count data (i.e., includes abundance information), revealed sample clustering based on sex, with male samples clustering more tightly than females. Permutational multivariate analysis of variance (PERMANOVA) revealed sex (male and female) had a significant impact (*p* = 0.001, 999 permutations) on the microbial diversity, though it only accounted for 5.7% variation in composition. Couple pairing also had a significant effect (*p* = 0.001, 999 permutations) on overall microbial composition and accounted for 48.2% of the variation.

**Figure 3:**
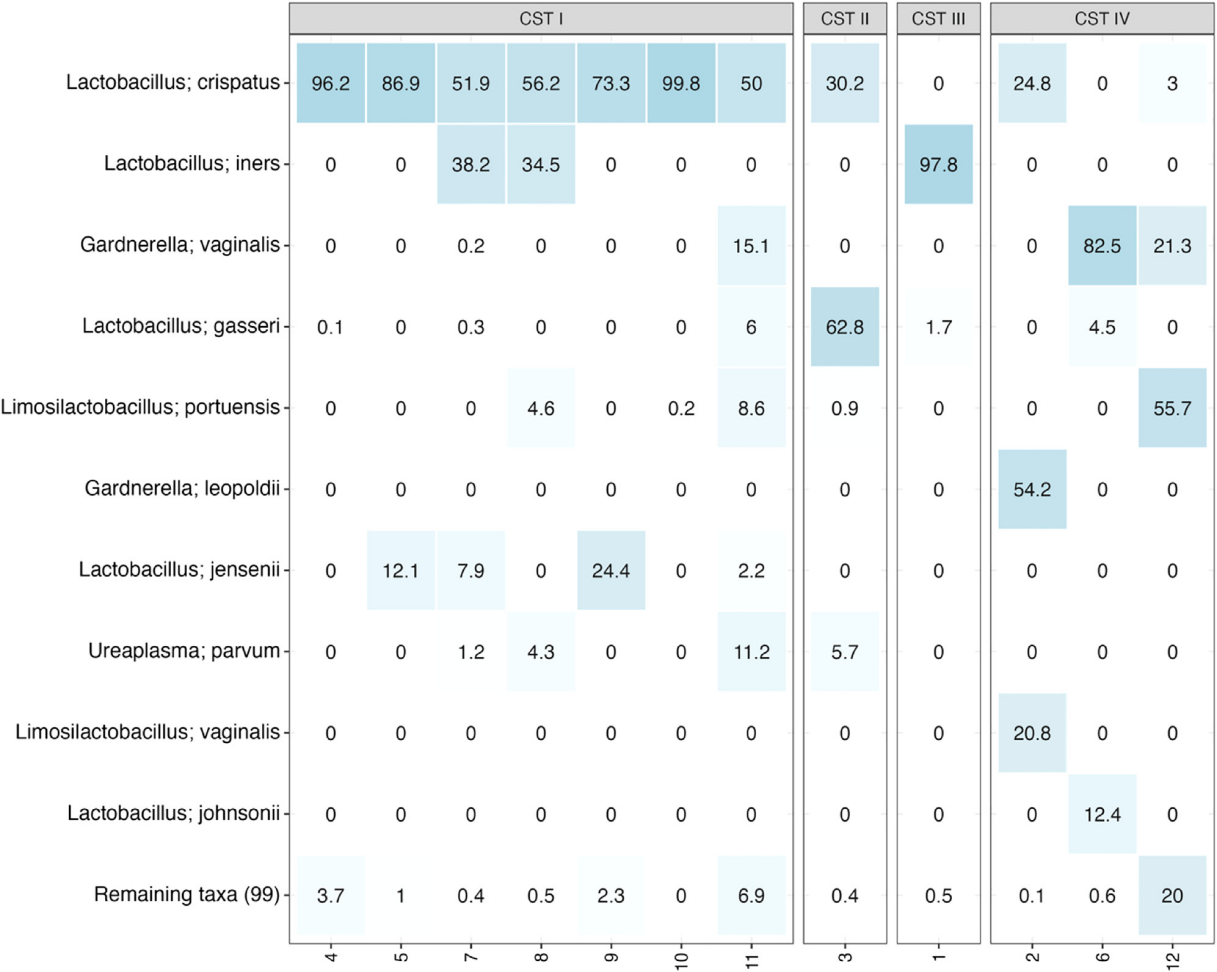
Taxonomic heatmap displaying relative abundance of species level composition of female *before* intercourse samples with vaginal community state type (CST) assignment

Female vaginal samples varied by both PCo1 and PCo2, showing a spread across both axes between other female vaginal samples. This is likely a reflection of the presence of different vaginal community state types (CSTs) between women in the study [Figure 3]. The vaginal microbiome consists of five main specific community state types (CSTs), with CSTs 1, 2, 3, and 5 each being dominated by one specific *Lactobacillus* spp. and 4 featuring low levels of *Lactobacillus* spp. and increased diversity of anaerobes such as *Gardnerella* spp. and *Mobiluncus* spp.^34^

**Figure 4:**
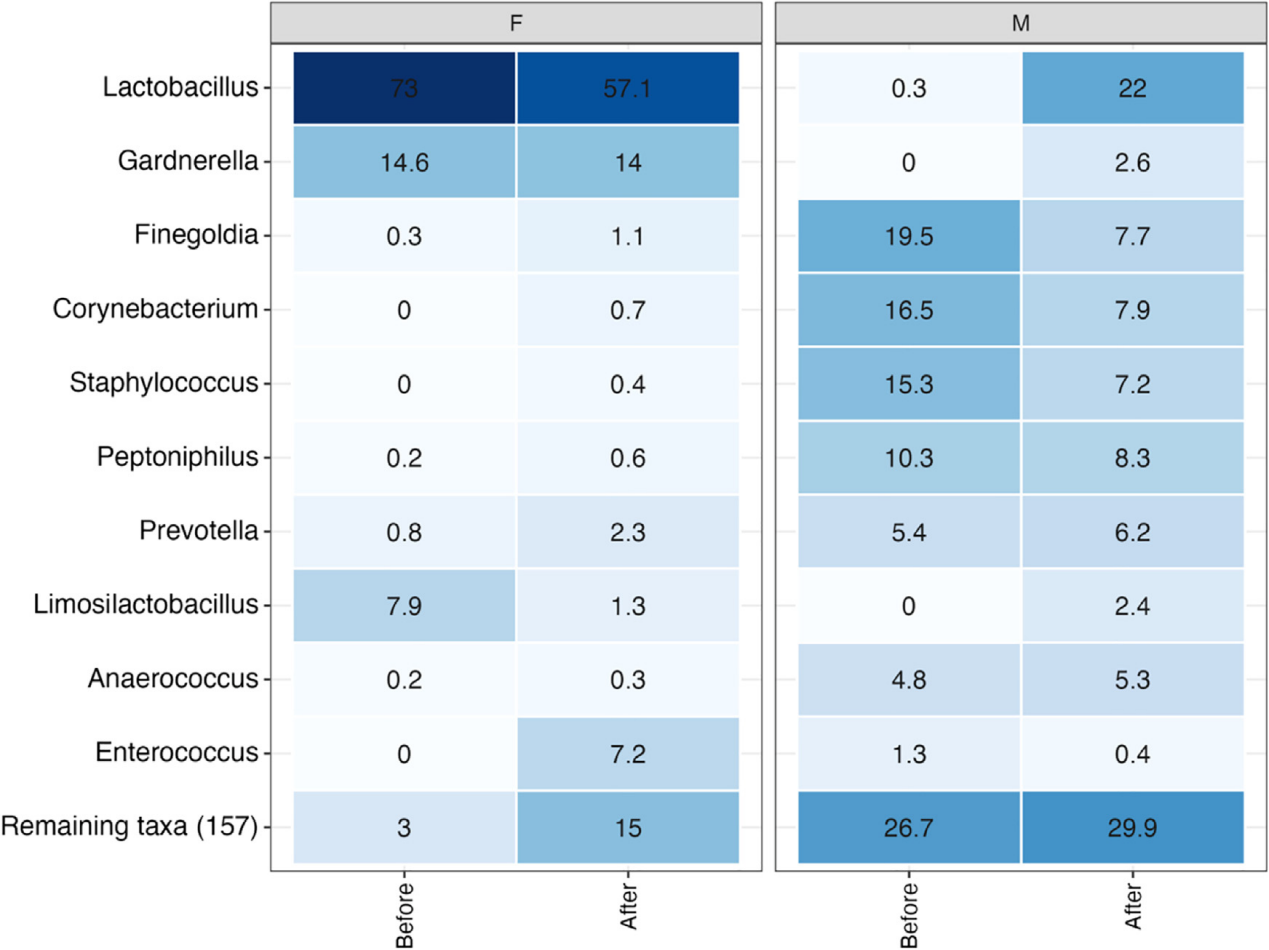
Heatmap displaying relative abundance of taxa grouping by sex and timepoint to show genera driving the disruption to microbial diversity in both vaginal and penile skin samples *after* intercourse

Overall, sample clustering based on couple was less apparent [Figure 2B]. There was an observable disruption to microbial diversity in both male and female samples between *before* and *after* sampling. Couples 1, 3, and 8 showed a distinct change in ordination between the male *before* and *after* sample. Here, the male *after* sample clustered closely with both female samples indicating male diversity becomes more homogeneous to female diversity *after* intercourse. Little change, however, was observed in female *before* and *after* samples. Couple 11 showed a similar trend, with female samples not undergoing a distinct change between sampling times whereas the male sample shifted toward more homogeneity with the female sample *after* intercourse, but not to the extent of that seen in couples 1, 3 and 8. Couples 2 and 9 showed the opposite trend occurring, where the male samples did not show a distinct change, and instead a distinct change in the female *after* sample was observed with the trajectory moving closer to that of the male partner. Minimal change was observed in couples 5 and 7 and couple 4 showed a distinct change with the trajectory of male and female *after* samples moving closer to each other, suggesting relatively equal donation of bacterial taxa from both sides. All samples from couples 6, 10, and 12 clustered together irrespective of sampling time. Couples 6 and 12 demonstrated tight clustering together for male and female samples both *before* and *after* intercourse, while couple 10 also displayed similar clustering of samples, however there was a similar change to that observed in couple 4, with the male and female *after* samples moving toward each other, but on a smaller scale. Couples 6, 10, and 12, which were most closely clustered in the PCoA, all engaged in intercourse without the use of a barrier contraceptive (i.e., condom), suggesting that unprotected vaginal intercourse promotes creation of a homogeneous bacterial community, between partners. It would be interesting to assess the longitudinal stability of this community after a period of prolonged abstinence to allow comment on whether male/female donation of bacteria is transient in nature or reflective of true colonization. Couples 10 and 12 had varying abstinence periods of two and four days respectively (no information for couple 6), however, durations of up to three weeks would likely be required for proper assessment given the well-documented changes in the vaginal microbiome that occur due to hormonal fluctuations in the menstrual cycle.^52^ To further investigate the bacterial taxa driving the changes in diversity observed, a heatmap was generated for males and females [Figure 4].

The heatmap shows an increase in the abundance of *Finegoldia*, *Corynebacterium*, *Staphylococcus*, *Peptoniphilus*, and *Enterococcus* in female samples *after* intercourse and a decrease of these genera in male samples following intercourse. An increase in abundance of *Limosilactobacillus* and *Lactobacillus* can be observed in male samples following intercourse with a decrease observed in female *after* samples. This same trend was observed when assessing transfer based on individual reads (data not shown). The introduced vaginal bacteria after intercourse are generally associated with the skin microbiome, likely introduced by the male. Taxa belonging to *Finegoldia*, *Corynebacterium*, *Staphylococcus*, *Peptoniphilus* and *Enterococcus* genera can be considered male contributors while *Limosilactobacillus* and *Lactobacillus* can be considered female contributors. The female vaginal microbiome is dynamic and influenced by factors such as age, ethnicity, pregnancy, menstrual cycle and diet.^53^ However, dominance of *Lactobacillus* is considered a hallmark of an ‘optimal’ vaginal microbiome. Taxa of the male penile microbiome has been more linked to common skin commensals such as *Corynebacterium*, *Staphylococcus, Finegoldia, Prevotella, Peptoniphilus*, *and Anaerococcus*.^36^^,^^54^ Genera were considered a contributor if the overall abundance decreased *after* intercourse and if the abundance in their partners sample increased *after* intercourse. We do acknowledge, however, that for true validation of microbial transfer, analyses would need to occur at a minimum of the serovar, but ideally, the strain level. At the present time serovar analysis using the full-length 16S rRNA gene has only been examined in *E. coli* and *Salmonella* spp., where it has shown some utility, but strain level analysis is not possible due to the limited genomic information this gene provides.^25^ Future studies will likely need to utilize either shotgun metagenomic sequencing of clinical samples or whole genome sequencing of individual bacterial isolates to achieve this level of taxonomic resolution.

### Microbiota transfer

For the purposes of exploring microbial transfer during intercourse for this dataset, a table detailing the total number of ASVs present in each participant and the transfer in each couple has been provided in Table 2. For each individual a baseline community was determined by the presence of an ASV in both *before* and *after* samples. ASVs that were identified as unique to an individual within the cohort were identified in order to assess unique and non-unique transfer during intercourse.

**Table 2:** Baseline total and unique ASV counts with microbial transfer observed across each couple in this dataset

Couple	ASVs Female			ASVs Male			Female to Male Transfer			Male to Female Transfer		
	Total	Unique	Proportion of Uniques (%)	Total	Unique	Proportion of Uniques (%)	Total	Unique	Proportion of Unique Transfer (%)	Total	Unique	Proportion of Unique Transfer (%)
1	3	0	0.0	3	2	66.7	1	0	0.0	1	1	100.0
2	3	2	66.7	12	5	41.7	2	1	50.0	0	0	NA
3	10	5	50.0	31	21	67.7	9	5	55.6	19	10	52.6
4	5	2	40.0	31	20	64.5	4	2	50.0	0	0	NA
5	5	2	40.0	53	37	69.8	2	0	0.0	5	2	40.0
6	4	3	75.0	28	10	35.7	1	1	100.0	14	5	35.7
7	16	7	43.8	36	31	86.1	3	0	0.0	3	3	100.0
8	8	1	12.5	27	15	55.6	8	1	12.5	2	1	50.0
9	2	1	50.0	46	34	73.9	2	1	50.0	29	22	75.9
10	1	1	100.0	11	4	36.4	2	1	50.0	0	0	NA
11	7	2	28.6	43	17	39.5	6	2	33.3	0	0	NA
12	15	8	53.3	42	11	26.2	7	6	85.7	3	2	66.7

Total ASV counts are determined by presence in both *before* and *after* samples.

A detailed analysis of two couples are presented within the main text [Figures 5 and 6] (data for remaining couples is available in the Figures S4–S13). Of the two couples presented here, one couple engaged in intercourse with the use of a condom (couple four) while the other did not (couple one).

**Figure 5:**
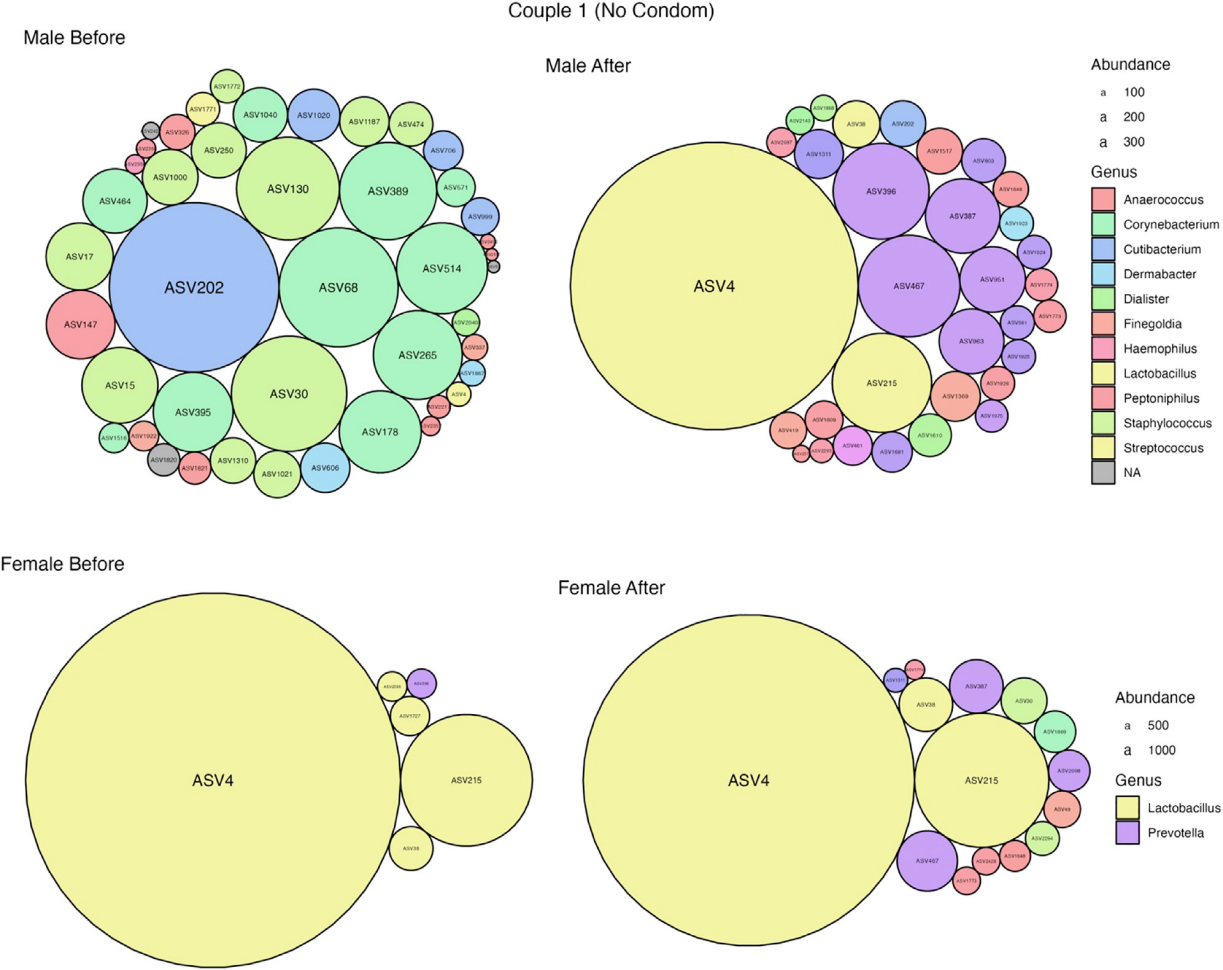
Taxonomic composition of the female vagina and male penile skin in couple 1 For each sample, taxonomy is visualized as circle packing. Each circle represents an ASV; relative abundance is proportional to the circle size and genus level taxonomy as color. Samples from left to right, top to bottom: male before, male after, female before and female after.

**Figure 6:**
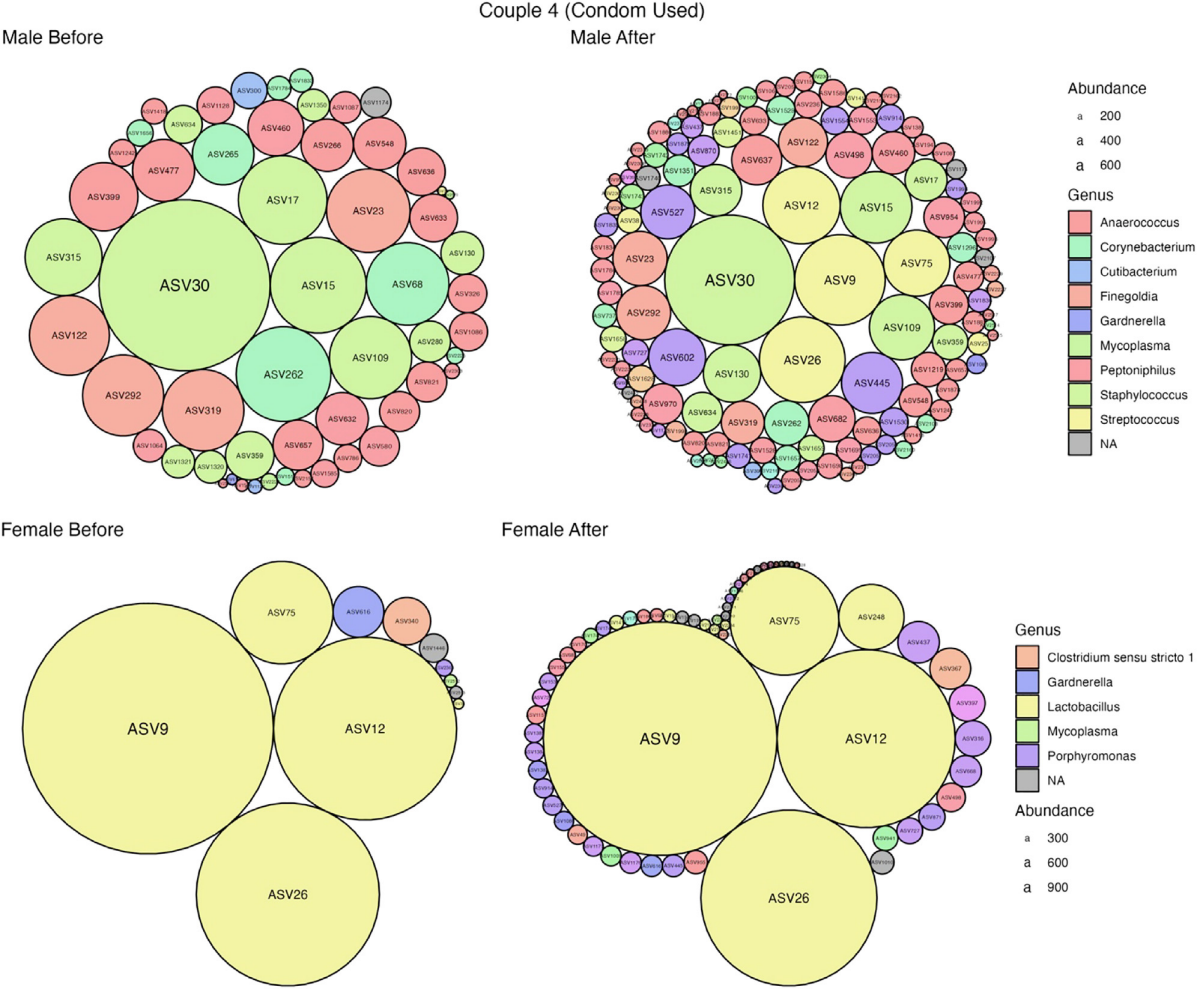
Taxonomic composition of the female vagina and male penile skin in couple 4 For each sample, taxonomy is visualized as circle packing. Each circle represents an ASV; relative abundance is proportional to the circle size and genus level taxonomy as color. Samples from left to right, top to bottom: male *before*, male *after*, female *before* and female *after*.

Prior to intercourse couple 1 had 44 and 6 ASVs for the male and female respectively [Figure 5]. This is consistent with current literature that states the male penile microbiome is more diverse than the female vaginal microbiome.^38^ Following intercourse*,* the number of ASVs detected in the male sample reduced to 32, while the number detected in the female more than doubled to 15. While there was a reduction in the total number of ASVs detected in the male sample *after* intercourse, three of the detected ASVs were new taxa, that were previously identified in the female *before* samples and classified as *Lactobacillus iners, Lactobacillus gasseri*, and *Prevotella corporis,* all of which are known to be associated with the human vagina.^55^^,^^56^ One of these ASVs was unique to the female in this cohort. These taxa are theorized to have transferred from the female to the male during sexual contact. Additionally, ASV4 identified as *Lactobacillus iners,* was detected in low abundance in the male *before* sample and high abundance in the female *before* sample. *After* intercourse, the abundance of ASV4 in the male sample increased to the most dominant ASV in the sample. The female sample *after* intercourse had a reduction in the abundance of ASV4 indicating likely transfer of this taxa from the female to the male during sexual contact. This is in line with a previous case report by Carda-Diéguez et al.^57^ Within this cohort, ASV4 was not detected in any other male during *before* sampling. This couple reported abstaining from intercourse for 5 days, suggesting it may be residual from a previous sexual encounter, or alternatively could be a commensal penile microbe, as has been previously reported.^58^^,^^59^ Assuming the male had showered in this time, it shows promise that bacterial signatures left on a perpetrator after an assault could still be detected several days later and despite maintaining standard hygiene practices. This also shows promise for development of a method for testing a suspected perpetrator post-assault as current STR typing relies solely on the detection of male DNA from the female victim.^10^ In this couple there was only one ASV transferred from the male to the female, and this was identified as *Staphylococcus epidermidis*. This ASV was not unique to the male of this couple. In the *before* male sample, taxa belonging to the *Corynebacterium* genus accounted for more than 20% of all taxa. *After* intercourse, there was a complete loss of this genus in the male sample. *Corynebacterium* is a well-documented penile coloniser but may be sensitive to disruption due to intercourse.^39^

The total ASV counts in couple 4 prior to intercourse were 56 and 11 for the male and female respectively [Figure 6]. *After* intercourse occurred, the number of ASVs detected in the male sample more than doubled (*n* = 133) while the number detected in the female was more than five and a half times more (*n* = 63) than detected *before* intercourse. Five of the new ASVs found in the male *after* samples were identified in the female *before* sample and identified as *Lactobacillus crispatus* (*n* = 4) and *Lactobacillus gasseri* (*n* = 1). These are theorized to have transferred from the female to the male during sexual contact. In both the male and female *after* sample, there was an introduction of ASVs (*n* = 19) that were not found in either male or female samples *before* intercourse occurred. These taxa are typically found in the gut, vagina, skin and oral microbiomes in humans^60^^,^^61^ and therefore suggest they have been introduced from other areas of the body during intercourse and/or through application of the condom. No ASVs were introduced to the female by the male in this couple. These results not only provide evidence of detectable microbial transfer occurring with the use of a condom but also provide evidence of introduced bacteria. The joint appearance of bacterial taxa in male and female samples taken *after* intercourse could be used as a marker for detecting sexual contact.

**Figure 7:**
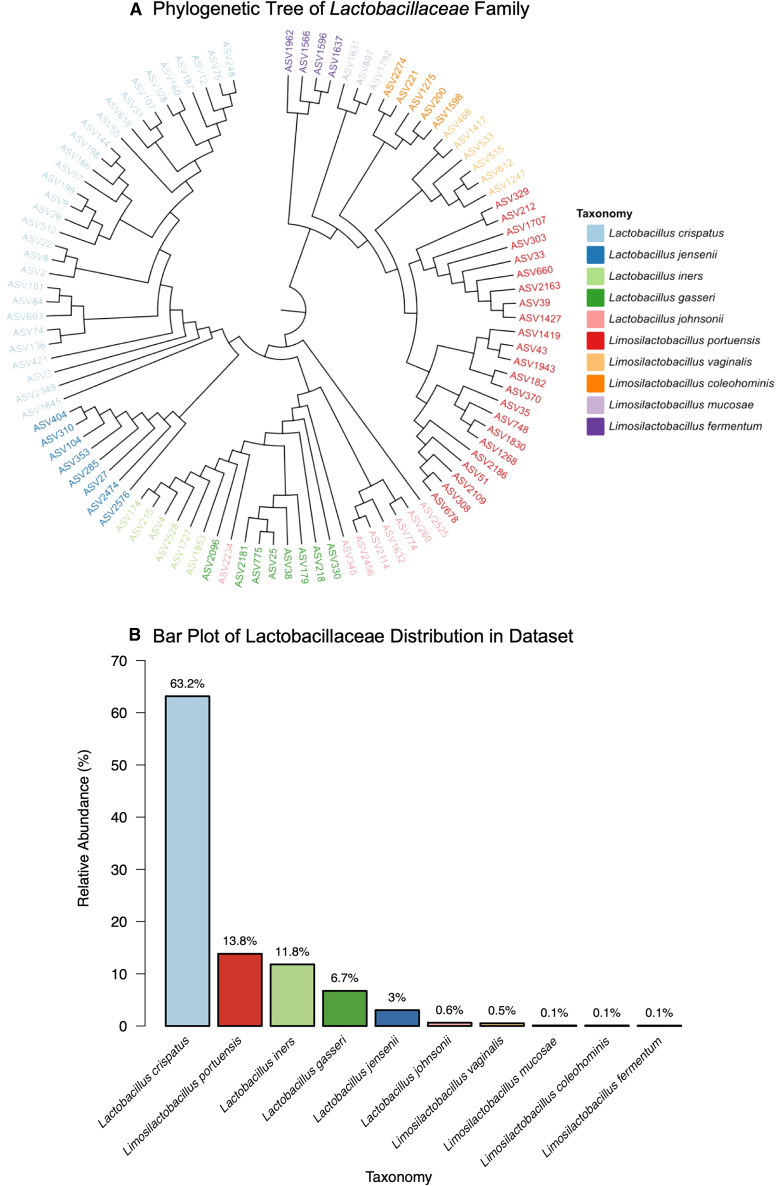
Distribution of *Lactobacillaceae* family (A) Phylogenetic tree displaying ASVs of *Lactobacillaceae* family. Sequence identity of ASVs were confirmed using BLAST against NCBI nucleotide data. (B) Bar plot displaying relative abundances of *Lactobacillaceae* family across all penile and vaginal samples. *Lactobacillus crispatus* accounted for more than 60% of the total *Lactobacillus* content across samples.

*Lactobacillus* spp. are common commensal vaginal bacteria considered to be markers of a healthy vaginal microbiome.^53^ The present study has found that when microbial transfer occurs between a male and female during intercourse, *Lactobacillus* spp. from the female contribute the highest amount. This is not surprising considering that of the five documented vaginal CSTs, four are dominated by a specific *Lactobacillus* spp. The presence of these *Lactobacillus*-dominated CSTs, however, varies among women depending on ethnicity and various lifestyle factors, especially smoking and cleansing of the vagina through a process known as douching. Whether or not there are CSTs that appear to ‘protect’ against male transfer while others are more receptive is yet to be determined. A larger sample number is needed to evaluate whether CST profiles had any relationship to the level of transfer observed in each pairing. Compared to females, males do not have a genera that is consistently present in almost all samples in high (greater than 80%) abundance.^54^ Future studies should therefore aim to target taxa belonging to the *Lactobacillus* genus when exploring forensic casework applications. This study found a total of 101 ASVs belonging to the *Lactobacillaceae* family [Figure 7A]. Just under 50% (*n* = 47) of those ASVs were only detected in one sample across the entire cohort and could be unique to those individuals they are detected in. In order to say this with any certainty, a much larger cohort would need to be analyzed and strain level variation observed. It does however show promise of interpersonal variation between individuals. *Lactobacillus crispatus* contributed to 63.2% of the total *Lactobacillaceae* content in penile and vaginal samples followed by *Limosilactobacillus* *portuensis* at 13.8%, *Lactobacillus iners* at 11.8% and *Lactobacillus gasseri* at 6.7% [Figure 7B].

### External factors and their impact on microbial diversity

As the application of the sexome in sexual assault casework is still in its infancy, it is important to completely understand the external factors that may have an impact on the microbial diversity of both males and females. Some of these include the use of a condom, lubricants, contact through digital, oral and anal intercourse, abstinence period and the presence of pubic hair. To observe the impact of condoms use on microbial diversity, alpha diversity plots using the Shannon diversity measure was used [Table S3]. Couple 3 was removed from this analysis as the participants listed the partial use of a condom (i.e., started without). Three of the 11 couples indicated that they used condoms during sexual intercourse. Analysis of communities *before* intercourse and grouped by condom use showed there was no significant difference in either male or female samples. Condom usage had a significant effect on the male diversity *after* intercourse (Shannon diversity measure, *p* = 0.018). In females, condom usage had no significant effect on the diversity *after* intercourse [Table S3]. These results validate that the use of a condom does impact microbial diversity and transfer. This is not to say that the use of a condom inhibits transfer of bacteria entirely though. Figure 6 shows that transfer of taxa does still occur despite condom use; however, the level of transfer and the taxa driving these differences may be impacted when compared to couples who did not use a condom. In addition, the male sampling technique used in this study involved penis shaft and glans sampling. This may have captured bacteria from the base of the penis shaft which was not entirely covered by the condom during the contact and therefore allowed for transfer from the female to the male.

In this study three of the 12 males indicated that they were circumcised. Comparison of alpha diversity showed no difference in *before* samples between circumcised and uncircumcised male samples [Table S3]. This is in marked contrast to the majority of studies that have previously compared the penile microbiota of circumcised and uncircumcised men,^38^ however our small sample size, is likely to have greatly impacted our ability to assess this. Of interest, though the only study which has reported no difference in penile microbiota between circumcised and uncircumcised men, Zozaya et al.,^42^ also used a similar sampling technique to our study, whereas most other studies sample either the coronal sulcus or meatus. In addition, male circumcision status did not significantly affect the diversity of vaginal samples taken from their female partner *after* intercourse [Table S3].

The presence or absence of pubic hair was also investigated to identify if there were any impacts to the microbiome. The presence of public hair did not have a significant effect on female diversity *before* intercourse [Table S3]. This is when observing the low vagina as opposed to the vulva which may have more contact with the pubic hair. While studies on this are limited, our finding is consistent with recent literature.^62^ As all males in this study (with the exception of one) had public hair present, a comparison in males was not possible.

### Conclusion

#### Casework applications

The data presented in this article demonstrate a disruption to the reproductive microbiome of the penis and vagina following penetrative sexual contact, however, this was not consistent across all couples. Further investigation of microbial diversity identified microbial ASVs that are unique to the individuals in this cohort (*n* = 24 participants). These unique ASVs were then identified in the individual’s sexual partner following intercourse, suggesting there has been a transfer of their unique bacterial signature during the contact. A transfer of non-unique ASVs was also observed in all couples. *Lactobacillus* spp. contributed to the most transfer from female to male and should be investigated as a potential target for future studies. The use of a barrier contraceptive (condom), occurrence of oral intercourse, lubricant use, circumcision status and presence of pubic hair did not have a statistically significant impact on the microbial diversity of males or females. While condom use did not have a statistically significant impact on microbial diversity, further investigation of transfer at an ASV level showed that when a condom was used the majority of transfer occurred from the female to the male. This shows promise for a means of testing a perpetrator post-assault and means there may be microbial markers that detect sexual contact even when a condom was used. The results of this research show that microbiome analysis may have some utility in investigation of sexual assault cases as an additional tool when male DNA is not present for traditional STR profiling to occur; however, substantial additional research is required to assess the reproducibility of within-couple results and assess whether higher level taxonomic classification (serovar level) is possible using a 16S rRNA gene approach for relevant bacteria. The use of shotgun metagenomics should also be considered, as this is likely to be more appropriate, considering its ability to uncover strain-strain transfer based on complete bacterial genomes. Consideration to human host DNA as needed in shotgun approaches as high abundance, particularly of female cells, will provide challenges for accurate on-target bacterial sequencing.^63^

Current protocols for the investigation of sexual assault which involve the isolation of male sperm cells from vaginal swabs indicate a signification reduction in the proportion of sperm-positive vaginal swabs when taken 48 h following an assault.^7^ While further exploration is required to adequately assess the persistence of the penile microbiome in the vagina post-intercourse, the data observed in this study suggests that microbiome analysis could allow the sampling window to be increased following an assault beyond what is currently possible with STR typing. However, there are numerous confounders at play, in particular in relation to the level of male personal hygiene implemented post-intercourse.

### Limitations of the study

Variability in the female vaginal microbiome has been observed during menses.^52^^,^^64^ While the present study aimed to control for this variation by requiring participants to sample at least 3–4 days *after* menses, further studies investigating the variability of bacteria during the complete menstrual cycle and the impact of disruption due to sexual contact are required. In order to identify unique bacterial strains with any certainty, whole genome sequencing must be used to analyze a larger cohort size of both males and females. The re-establishment of baseline vaginal and penile microbiomes following disruption due to sexual intercourse and the persistence of the sexome following sexual intercourse has not yet been investigated and should be a focus of future studies.

## Resource availability

### Lead contact

Requests for further information and resources should be directed to and will be fulfilled by the lead contact, Ruby Dixon (ruby.dixon@murdoch.edu.au).

### Materials availability

This study did not generate new unique reagents.

### Data and code availability

• Data- Supporting data is available at https://github.com/ruby-dixon/PacBio-Sexome.
• Code- Supporting code is available at https://github.com/ruby-dixon/PacBio-Sexome.
• Additional information- Any additional information required to reanalyze the data reported in this paper is available from the lead contact upon request.

## Acknowledgments

This study was funded and supported by the Murdoch University Bachelor of Science Honours program under the school of Medical, Molecular and Forensic Sciences.

## Author contributions

Conceptualization, B.C., S.E., and R.D.; methodology, B.C., S.E., and R.D.; investigation, R.D., and C.M.; data curation, R.D., and S.E.; writing – original draft, R.D.; writing – review and editing, R.D., B.C., S.E., C.M., and M.P.; visualization, R.D.; supervision, B.C. and S.E.

## Declaration of interests

The authors declare no competing interests.

## STAR★Methods

### Key resources table

**Table:** 

REAGENT or RESOURCE	SOURCE	IDENTIFIER
**Bacterial and virus strains**		

ZymoBIOMICS™ Microbial Community Standard	Zymo Research, USA	D6300

**Deposited data**		

https://github.com/ruby-dixon/PacBio-Sexome.	N/A	N/A

**Oligonucleotides**		

27F (5′-AGRGTTYGATYMTGGCTCAG-3′)		
Integrated DNA Technologies		
UNITAG-F (GCAGTCGAACATGTAGCTGACTCAGGTCAC)		
UNITAG-R (TGGATCACTTGTGCAAGCATCACATCGTAG)		

**Software and algorithms**		

RStudio (v1.4) and R (v4.1.1)		
*phyloseq* (v1.38.0)		
*ape* (v5.7.1)		
*Biostrings* (v2.62.0)		
*decontam* (v1.14.0)		
*microbiomeutilities* (v1.00.16)		
*ampvis2* (v2.8.6)		
*ggplot2* (v3.4.1)		
*packcircles* (v0.3.5)		

### Experimental model and study participant details

#### Participant recruitment and selection

Human vaginal and penile skin swabs were collected from consensual male/female couples who were self-reported as healthy, with no history of sexually transmitted infections or reproductive medical conditions. Each couple was required to be in a long-term monogamous relationship (>12 months) with both participants above 19 years of age. These inclusion criteria were decided in conjunction with the lead organisation’s Human Research Ethics Committee to mitigate risk to participants. Each male and female volunteer provided written consent to participate in the study, which was approved by the relevant Human Research Ethics Committee (Protocol 2020/059). Recruitment occurred over two cohorts, the first conducted in 2020 and the second in 2021. The recruitment cohorts resulted in samples from seven and five male-female pairings, respectively, totaling 12 couples.

#### Human ethics

This research was conducted using human participants with informed consent. Research was approved by the Murdoch University Human Research Ethics Committee (Protocol 2020/059).

### Method details

#### Collection of intimate samples

Samples from the genital region were self-collected by each volunteer participant in a male and female pairing. Female participants used sterile Copan® rayon swabs to self-collect samples, following the technique for low vaginal swab (LVS) self-sampling by PathWest Laboratory Medicine W.A.^65^ [Figure S1]. The method for male sampling followed a process of swabbing up and down the penis shaft and around the glans of the penis using the same type of Copan® swab, moistened with sterile water. Details of this procedure are described in the volunteer information pack [Figure S2]. *Before* intercourse samples were collected after at least two to four days of abstinence from sexual intercourse. In addition, females were asked to complete the *before* sample collection at least three to four days *after* menses to control against cyclic variation in the vaginal microenvironment associated with menstruation.^66^ Five replicate swabs were collected for each participant and timepoint, totaling 20 per couple (10 male, 10 female). Participants were asked to collect *after* intercourse samples within 3 – 12 hours after penetrative intercourse to reflect a practicable and realistic window for medical examination post-sexual assault in casework. Volunteers returned their kits to a nominated third party, who stored samples in a locked -20°C freezer until processing.

#### Mock Community

The ZymoBIOMICS™ Microbial Community Standard (Zymo Research, USA) (catalogue #: D6300) was included as a positive control to evaluate the extraction protocol. This whole cell mix consists of 10 inactivated microorganisms - eight phylogenetically distant bacteria including both Gram positive and negative cell structure and two fungi which were not amplified in this protocol (Table S1).

#### DNA extraction and QC

DNA from bacterial communities was extracted using the PureLink™ Microbiome DNA Purification Kit (Invitrogen™, USA) as described in the user guide for buccal, vaginal and skin swab samples.^67^ During the lysis step, samples underwent a bead beat mechanical lysis using horizontal agitation. This was performed on a TissueLyser II (Qiagen®, Germany) at 25 Hz for 2 × 3 minutes. Penile skin (*n* = 120) and vaginal swab samples (*n* = 120) were processed in batches by couple to reduce the risk of cross-contamination. Extraction control blanks (*n* = 12) were processed with each batch to assess background microbe levels^68^^,^^69^ and for quality control. For continuity, the extraction control blanks contained a sterile Copan® rayon swab identical to those used to collect the intimate samples. DNA from samples were eluted in 50μL of elution buffer and stored at -20°C prior to further analysis.

Sample quality control was performed on all samples to ensure replicate swabs with the highest bacterial template were used for subsequent library preparation. Purified DNA samples underwent PCR to amplify the 16S rRNA gene hypervariable V4 region with primers 515F and 806R.^70^ Amplified PCR products were run on agarose gels to assess concentration and successful amplification of the bacterial target. Those DNA extracts with the highest amplicon concentration observed by gel electrophoresis were progressed to high-throughput sequencing, totaling 24 male and 24 female samples, alongside extraction control blanks, and one positive control (ZymoBIOMICS Mock Community).

#### Gene amplification and barcoding

Purified DNA extracted from vaginal and penile skin swab samples underwent PCR to amplify the full-length 16S rRNA gene using primers 27F (5′-AGRGTTYGATYMTGGCTCAG-3′) and 1492R (5′-RGYTACCTTGTTACGACTT-3′), along with universal sequences UNITAG-F (GCAGTCGAACATGTAGCTGACTCAGGTCAC) and UNITAG-R (TGGATCACTTGTGCAAGCATCACATCGTAG) and amine blocks incorporated at the 5′ end of each primer.^71^ This was to ensure that unbarcoded amplicons carried over from the PCR did not undergo DNA ligation with SMRTbell adaptors during sequencing library preparation. To obtain 16S rRNA gene amplicons, a two-step library preparation method was used. First round PCR was carried out in 30 μL reactions containing 0.3 μM each of the forward and reverse primers, 15 μL of 1X AccuStart™ II ToughMix® (Quantabio™, USA), 0.75 μL each of ArcticZymes dsDNase and DTT, 6.6 μL nuclease-free water and 6 μL of purified DNA template or nuclease-free water (negative control). Two negative template controls were included. The dsDNase and DTT were included as part of the PCR Decontamination Kit™ (ArcticZymes Technologies, Norway), which was used to remove any contaminating DNA in the PCR master mix without reduction of PCR sensitivity.^68^ The dsDNase activation was achieved by incubation of the master mix at 37°C for 20 minutes in the absence of template DNA. The dsDNase was then inactivated by incubation at 60°C for 20 minutes. This ensured that the template added after inactivation remained safe from digestion. The first round PCR was performed with cycling conditions as follows: initial denaturation at 94°C for 3 minutes, followed by 30 cycles at 94°C for 30 secs, 52°C for 30 secs, and 72°C for 2 minutes and a final extension step of 72°C for 5 minutes. PCR products were then purified using NucleoMag NGS magnetic beads (Macherey-Nagel™, Germany) at a 0.6X ratio and eluted in 30 μL low Tris-EDTA (TE) buffer. To confirm the presence and appropriate size of amplicons, PCR products were visualized on a QIAXcel® automated gel electrophoresis system (Qiagen®). Amplicons were normalized to 1 ng/μL prior to the second round barcoding PCR for individual sample indexing. These were carried out in 25 μL reactions containing 0.3 μM each of the unique forward and reverse barcoded primers, 12.5 μL of 1X AccuStart™ II ToughMix® (Quantabio™), 3 μL nuclease-free water and 2 μL of template or nuclease-free water from first round PCR. PCR cycling conditions were the same as detailed in the first round PCR, but reduced to 8 cycles.

#### PacBio sequencing

To accurately identify bacterial taxa beyond genus level, resolution beyond V3-V4 regions of the 16s rRNA gene is needed. PacBio long-read bacterial 16S rRNA gene sequencing was undertaken to analyse the full V1 – V9 region. Barcoded second round PCR products were pooled in equimolar concentrations based on QIAxcel® (Qiagen®) quantification of the target ∼1500 bp band. Amplicon pools were purified using NucleoMag® NGS magnetic beads (Macherey-Nagel™) at a 0.55X ratio and eluted into 50 μL low TE buffer. Pools were normalised to ∼500 ng of DNA in 37 μL of low TE. Normalised amplicon pools were provided to the Australian Genome Research Facility (AGRF) sequencing service where SMRTbell adapters were ligated onto barcoded PCR products and sequenced on a single SMRT® cell using the PacBio® Sequel II System in accordance with manufacturer recommendations (AGRF service guide,^72^ Part no. 101-599-700, REV05, May 2022, Pacific Bioscience^73^). Circular consensus sequence (CCS) reads were generated from the raw PacBio sequencing data using the SMRTLink software (v 11.0.0.146107) provided by the manufacturer.

### Quantification and statistical analysis

#### Processing of HiFi read files

PacBio CSS .bam files were first demultiplexed using *lima* (v2.7.1) with the settings - hifi-preset ASYMMETRIC and then converted to FASTQ format using *pbtk* (v3.1.0) bam2fastq function. Sequence data was analysed using the *DADA2* (v1.22.0) pipeline.^74^ First, primers were removed, and sequences were quality filtered. The *DADA2* denoise algorithm was then used to produce amplicon sequence variants (ASVs).^75^ Taxonomy was assigned using the Bayesian classifier with reference to curated SILVA v128 database.^76^ Taxonomic species-level assignments were then confirmed for most abundant taxa belonging to *Lactobacillaceae* family with the SILVA v128 database. This was performed using BLAST analysis against the National Center for Biotechnology Information (NCBI) nucleotide (nt) database.^77^ ASVs were assigned to species where possible based on the top hit (highest percent identity and query coverage along with the lowest E value).

#### Data handling and analysis

RStudio (v1.4) and R version 4.1.1^78^ was used to analyse the data to assess trends in microbial community profiles across samples both within, and across, sexual partners. First, the *phyloseq* (v1.38.0) package was used to create a manageable object from the *DADA2* output containing ASV abundances, taxonomy table and sample metadata. Next, the *ape* (v5.7.1) and *Biostrings* (v2.62.0) packages were used to extract the phylogenetic tree and reference sequences which were then merged with the *phyloseq* object. Prior to analysis, the *decontam* (v1.14.0) package was used to identify contaminants in samples to produce more accurate profiling of the microbial communities.^79^ The prevalence method with a 0.05 probability threshold was set. This method compares the prevalence of ASVs between samples and negative controls to determine likely contaminants.^79^ The *microbiomeutilities* (v1.00.16), *ampvis2* (v2.8.6) and *ggplot2* (v3.4.1) packages were used to visualise the data. Alpha diversity measures were carried out to assess the richness and diversity of individual samples. Alpha diversity was measured using the Shannon diversity metric and the Wilcoxon test for significance.^33^ Beta diversity measures were carried out to assess the richness and diversity of samples across different communities. Beta diversity was measured using Principle Coordinates Analysis (PCoA) with Bray-Curtis^50^ distance measures. ASV's that were not present in more than 0.1% relative abundance in any sample were removed. A heatmap was used to observe microbial composition of aggregated female and male samples, *before* and *after* intercourse to determine taxa driving differences in diversity. The *packcircles* (v0.3.5) package was used to generate packing circles to explore microbial transfer between couples following intercourse. Permutational analyses of variance (PERMANOVA) were used to assess multivariate community level differences between males and females.^80^
