# Supplementary material for: Bacterial transfer during sexual intercourse as a tool for forensic detection
Source: iScience. 2025 Feb 12;28(2):111861. doi: 10.1016/j.isci.2025.111861 (PMC11889491; doi:10.1016/j.isci.2025.111861)
Supplement: Document S1. Figures S1–S13 and Tables S1–S4 [file mmc1.pdf]

iScience, Volume *28*

Supplemental information

Bacterial transfer during sexual intercourse as a tool for forensic detection

Ruby Dixon, Siobhon Egan, Matthew Payne, Christopher Mullally, and Brendan Chapman

Supplementary Information

Index

- **Fig S1**. Diagram of female self-collection of intimate swabs.
- **Fig S2**. Diagram of male self-collection of intimate swabs.
- **Fig S3:** Boxplot displaying relative abundance of bacteria identified in ZymoBIOMICS mock community positive control.
- **Fig S4:** Taxonomic composition of the female vagina and male penile skin in couple 2.
- **Fig S5:** Taxonomic composition of the female vagina and male penile skin in couple 3.
- **Fig S6:** Taxonomic composition of the female vagina and male penile skin in couple 5.
- **Fig S7:** Taxonomic composition of the female vagina and male penile skin in couple 6.
- **Fig S8:** Taxonomic composition of the female vagina and male penile skin in couple 7.
- **Fig S9:** Taxonomic composition of the female vagina and male penile skin in couple 8.
- **Fig S10:** Taxonomic composition of the female vagina and male penile skin in couple 9.
- **Fig S11:** Taxonomic composition of the female vagina and male penile skin in couple 10.
- **Fig S12:** Taxonomic composition of the female vagina and male penile skin in couple 11.
- **Fig S13:** Taxonomic composition of the female vagina and male penile skin in couple 12.
- **Table S1:** ZymoBIOMICS Microbial Community Standard theoretical 16S rRNA gene composition and NCBI accession numbers to reference genomes (81).
- **Table S2.** Participant demographics.
- **Table S3.** Table of results for alpha diversity plots between variables in the study.
- **Table S4.** Contaminants identified from the *decontam* package and removed from the data.

PARTICIPANT INSTRUCTIONS (FEMALE)

PLEASE NOTE: Samples are time sensitive and must be dropped off within 30 hours of collection.

PRIOR TO INTERCOURSE

1. **Wash your hands** thoroughly with soap and water and dry before taking a sample.
2. Find a comfortable position, either sitting or standing.
3. Remove the swab applicator labelled "BEFORE" from the packaging. **Avoid touching the cotton tip.**
    a. You are able to apply all swabs at once; or complete one at a time
4. Part the labia and put the applicator end (cotton tip) about 2cm (length of one finger joint) inside your vagina.
5. Gently turn the swab around once, then leave for a count of 10 seconds.
6. Remove the swab, being careful not to touch any other skin.
7. Place the swab directly into its container.
8. Wash your hands.
9. Place into plastic bag provided.

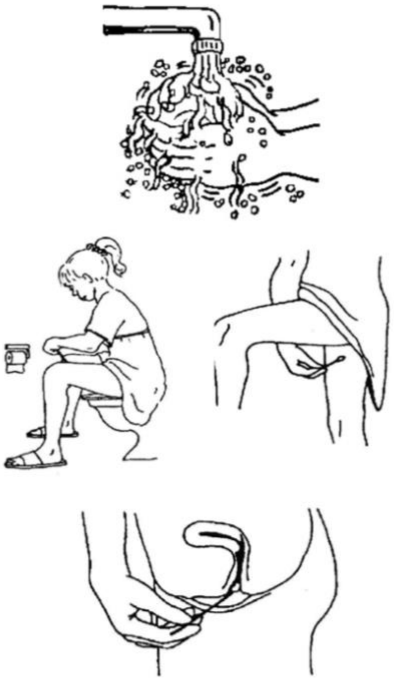

AFTER INTERCOURSE

10. Repeat steps 1 – 9 for swabs labelled "AFTER".
11. Store in refrigerator until delivery to (REDACTED). You may keep the sample at room temperature for up to 2hrs in order to transport it to campus.

Fig S1. Diagram of female self-collection of intimate swabs

PARTICIPANT INSTRUCTIONS (MALE)

PLEASE NOTE: Samples are time sensitive and must be dropped off within 24 hours of collection.

PRIOR TO INTERCOURSE

1. **Wash your hands** thoroughly with soap and water and dry before taking a sample.
2. Find a comfortable position, either sitting or standing.
3. Remove the swab applicator labelled “BEFORE” from the packaging**. Avoid touching the cotton tip**.
4. Moisten the swab with the distilled water provided.
    a. Please complete ONE swab at a time, moistening EACH swab with the distilled water provided.
    b. NOTE: Discard water after use.
5. As per the diagram below, swab up and down the penis shaft and around the glans (head) of the penis (if you are uncircumcised, you’ll need to retract the foreskin prior to doing this). Repeat this action 5 times.
6. Place the swab directly into its container.
7. Repeat steps 1 – 6 for all five swabs.
8. Wash your hands.
9. Place in the plastic bag provided.

AFTER INTERCOURSE

10. Repeat steps 1 – 9 for swabs labelled “AFTER”.
    a. NOTE: Discard water after use.
11. Place into plastic bag provided.
12. Store in refrigerator until delivery to (REDACTED). You may keep the sample at room temperature for up to 2hrs in order to transport it to campus.

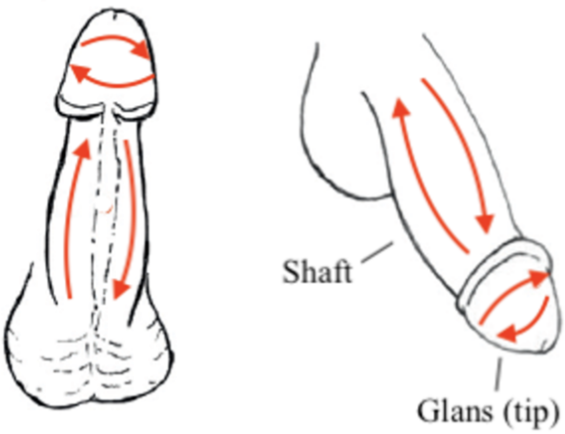

Fig S2. Diagram of male self-collection of intimate swabs

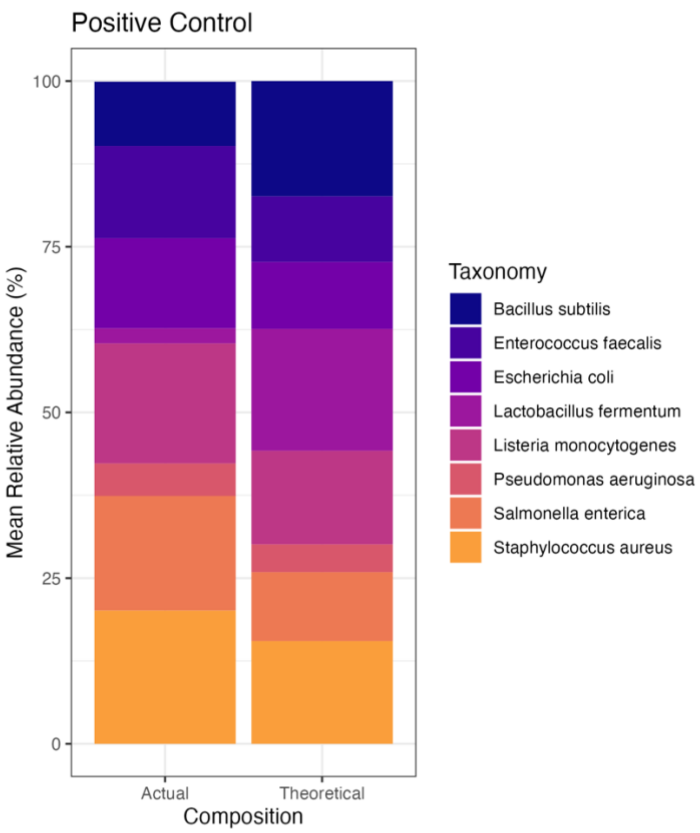

Fig S3: Boxplot displaying relative abundance of bacteria identified in ZymoBIOMICS mock community positive control. The theoretical composition of these eight bacteria are displayed as provided by manufacturer (Zymo Research), alongside data produced from this study.

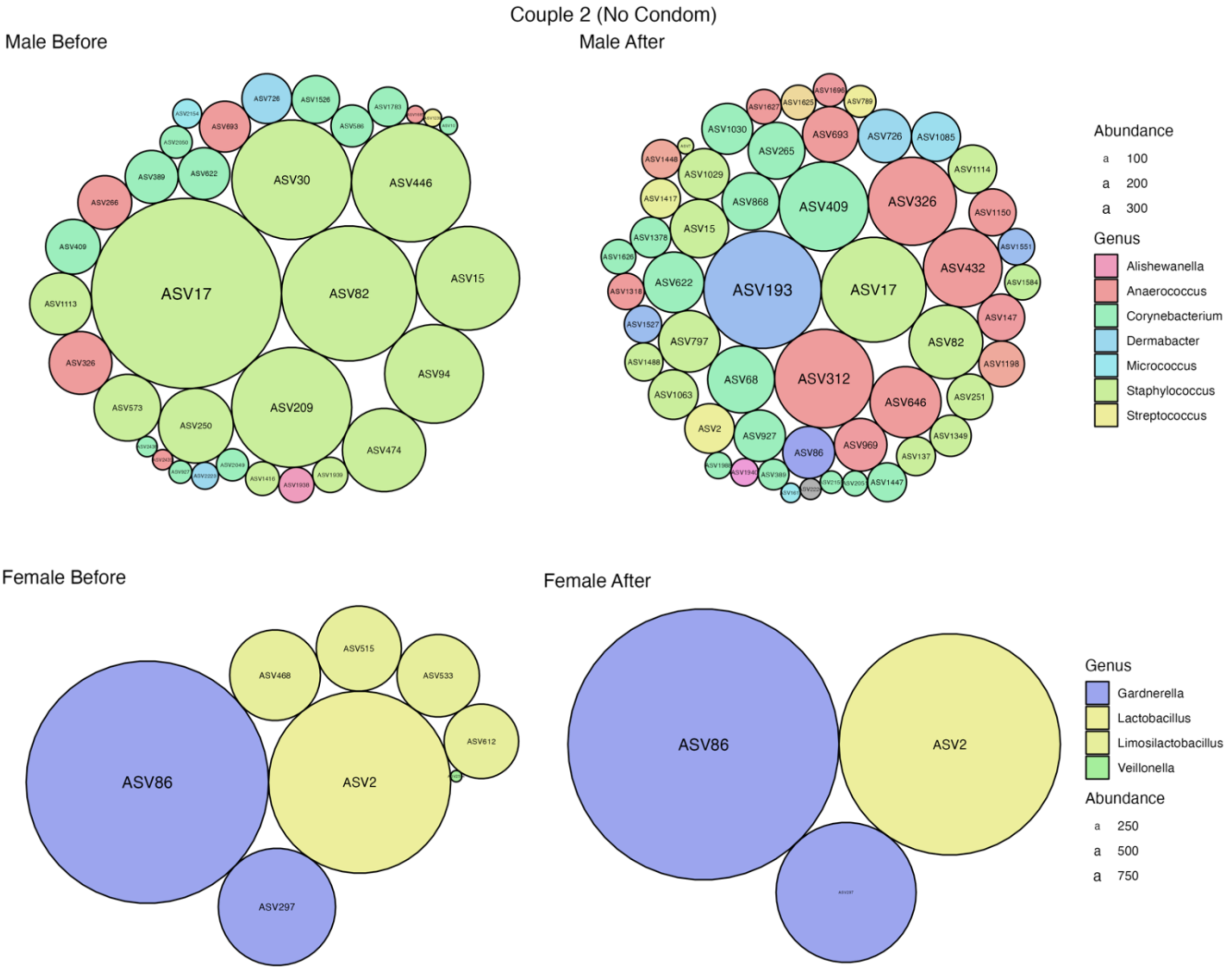

Fig S4: Taxonomic composition of the female vagina and male penile skin in couple 2. For each sample, taxonomy is visualized as circle packing. Each circle represents an ASV; relative abundance is proportional to the circle size and genus level taxonomy as colour. Samples from left to right, top to bottom: male *before*, male *after*, female *before* and female *after*.

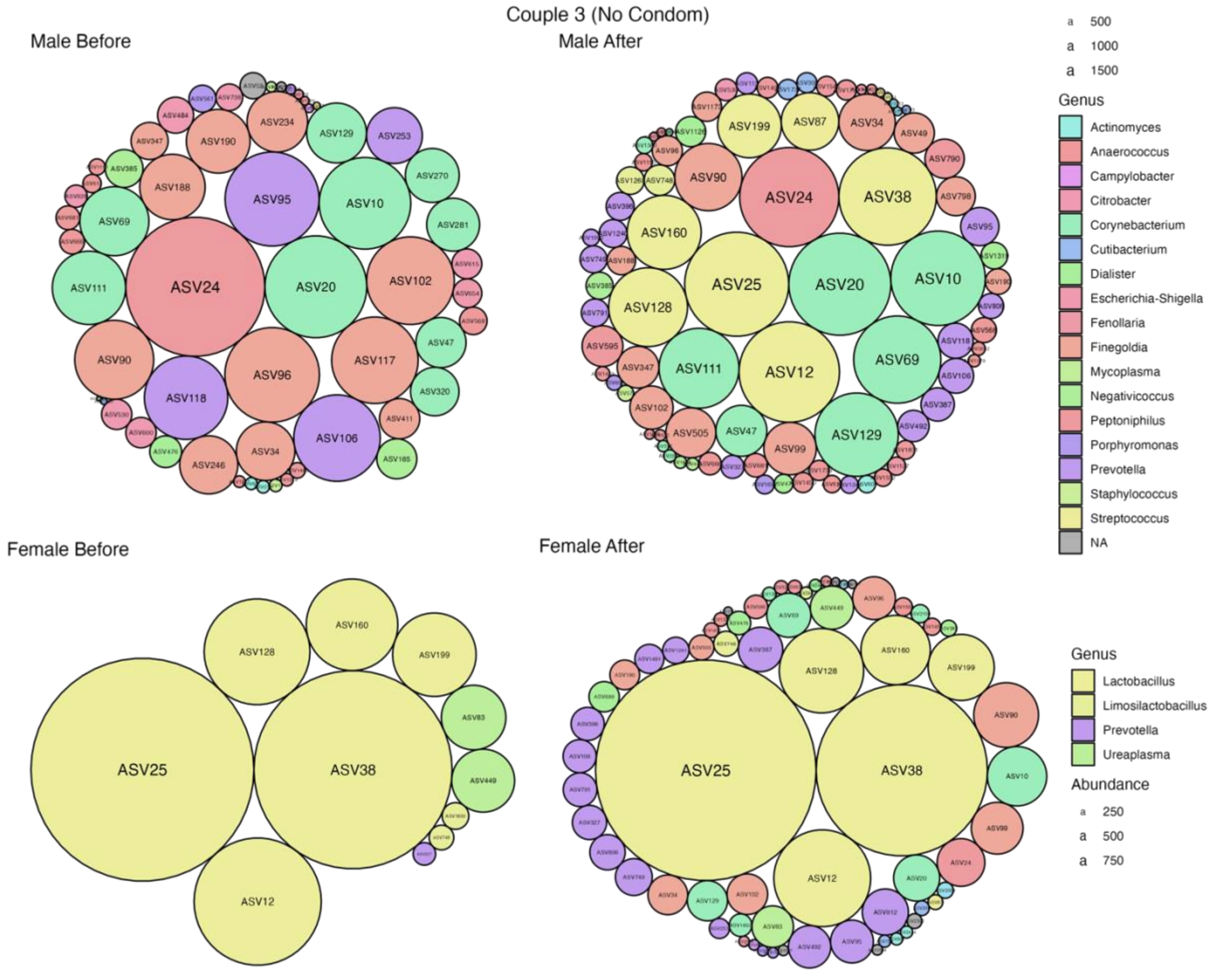

Fig S5: Taxonomic composition of the female vagina and male penile skin in couple 3. For each sample, taxonomy is visualized as circle packing. Each circle represents an ASV; relative abundance is proportional to the circle size and genus level taxonomy as colour. Samples from left to right, top to bottom: male *before*, male *after*, female *before* and female *after*.

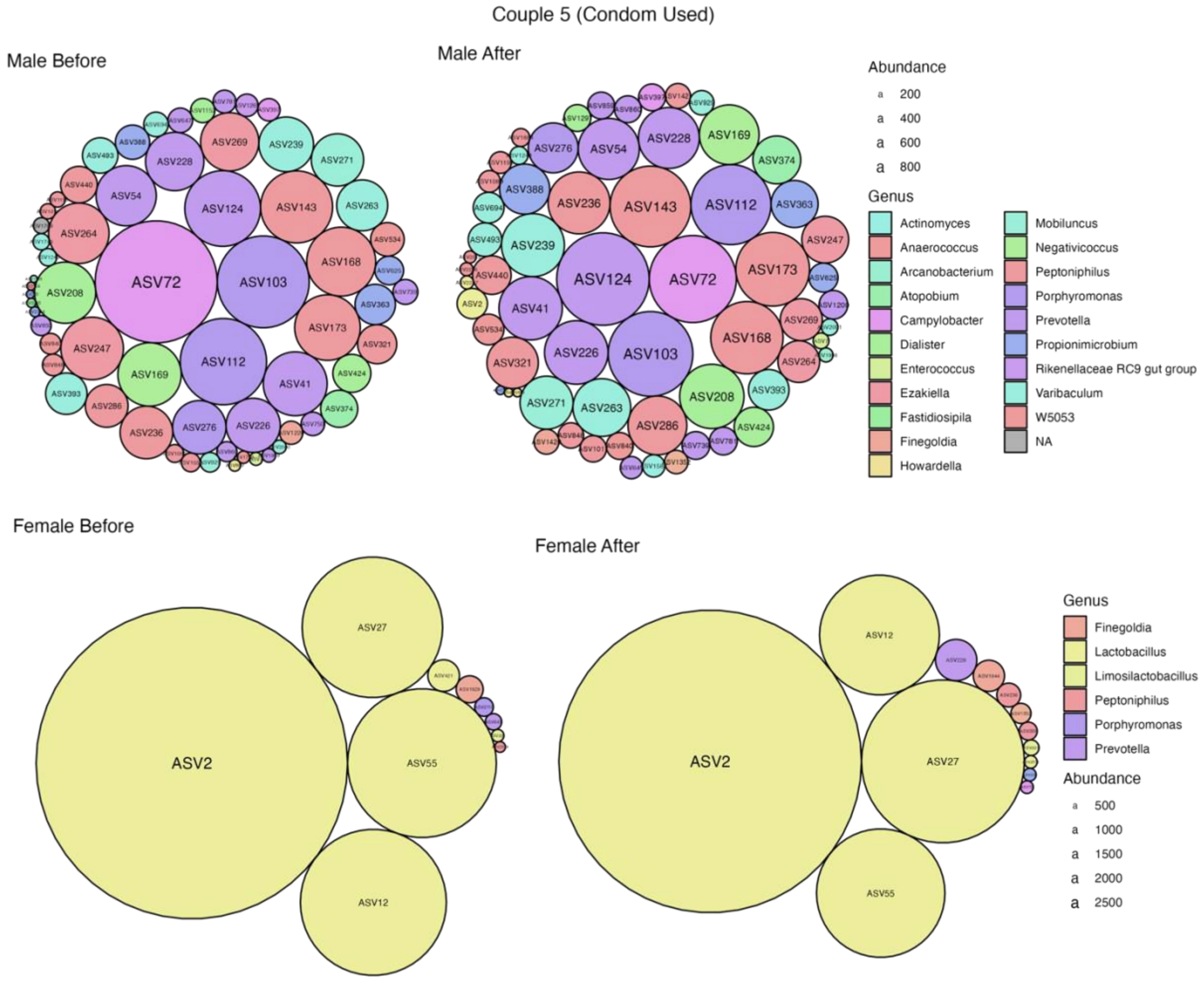

Fig S6: Taxonomic composition of the female vagina and male penile skin in couple 5. For each sample, taxonomy is visualized as circle packing. Each circle represents an ASV; relative abundance is proportional to the circle size and genus level taxonomy as colour. Samples from left to right, top to bottom: male *before*, male *after*, female *before* and female *after*.

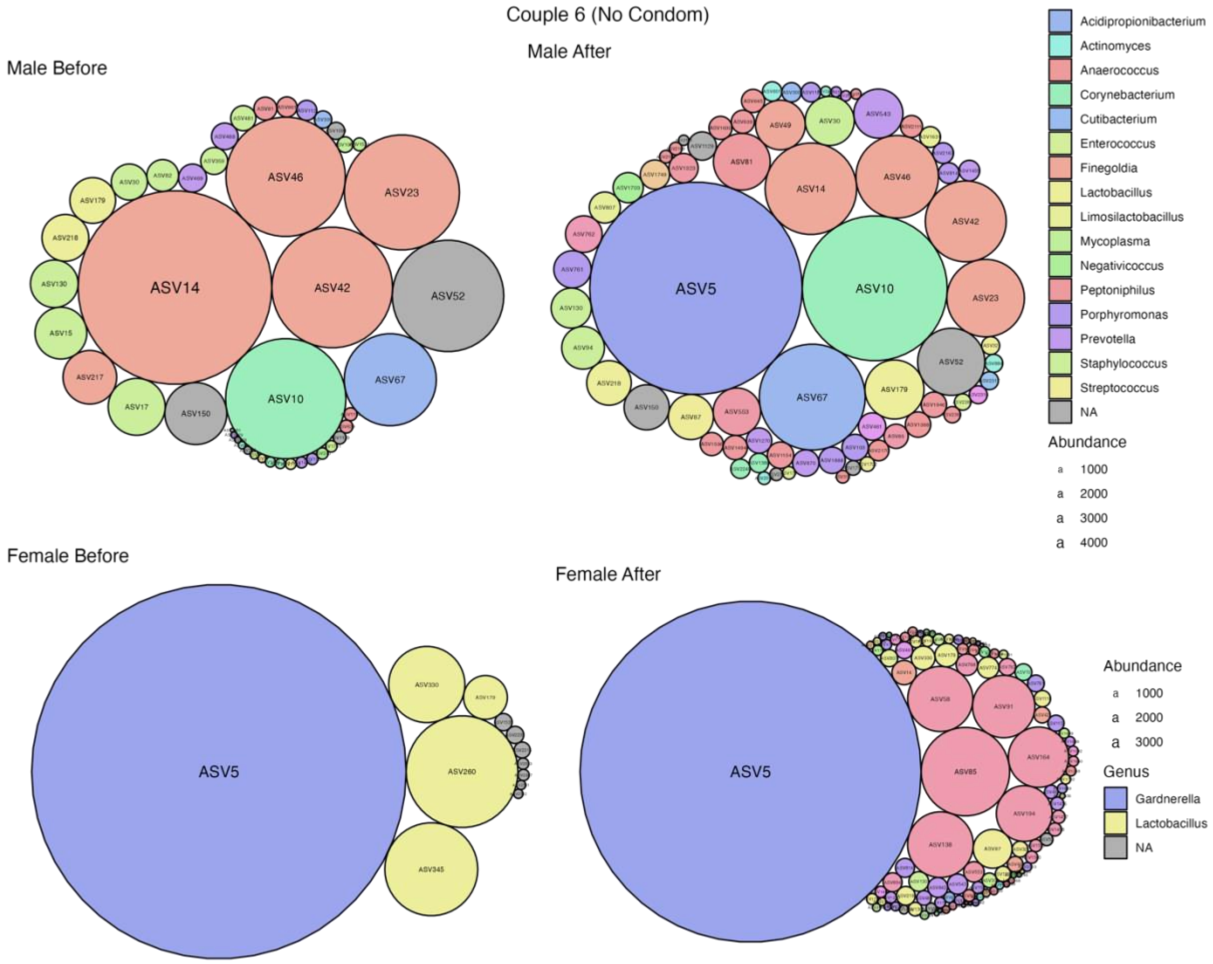

Fig S7: Taxonomic composition of the female vagina and male penile skin in couple 6. For each sample, taxonomy is visualized as circle packing. Each circle represents an ASV; relative abundance is proportional to the circle size and genus level taxonomy as colour. Samples from left to right, top to bottom: male *before*, male *after*, female *before* and female *after*.

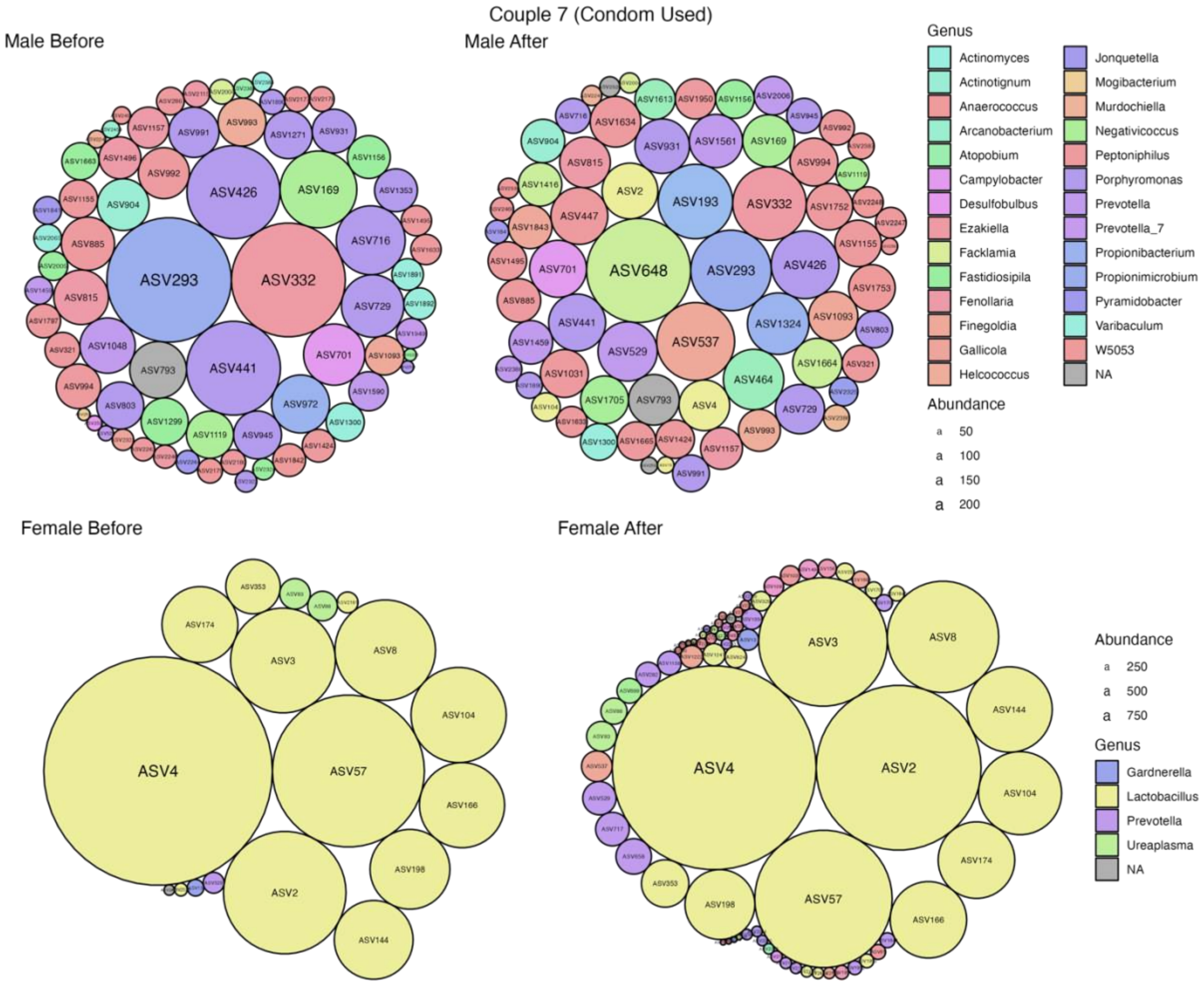

Fig S8: Taxonomic composition of the female vagina and male penile skin in couple 7. For each sample, taxonomy is visualized as circle packing. Each circle represents an ASV; relative abundance is proportional to the circle size and genus level taxonomy as colour. Samples from left to right, top to bottom: male *before*, male *after*, female *before* and female *after*.

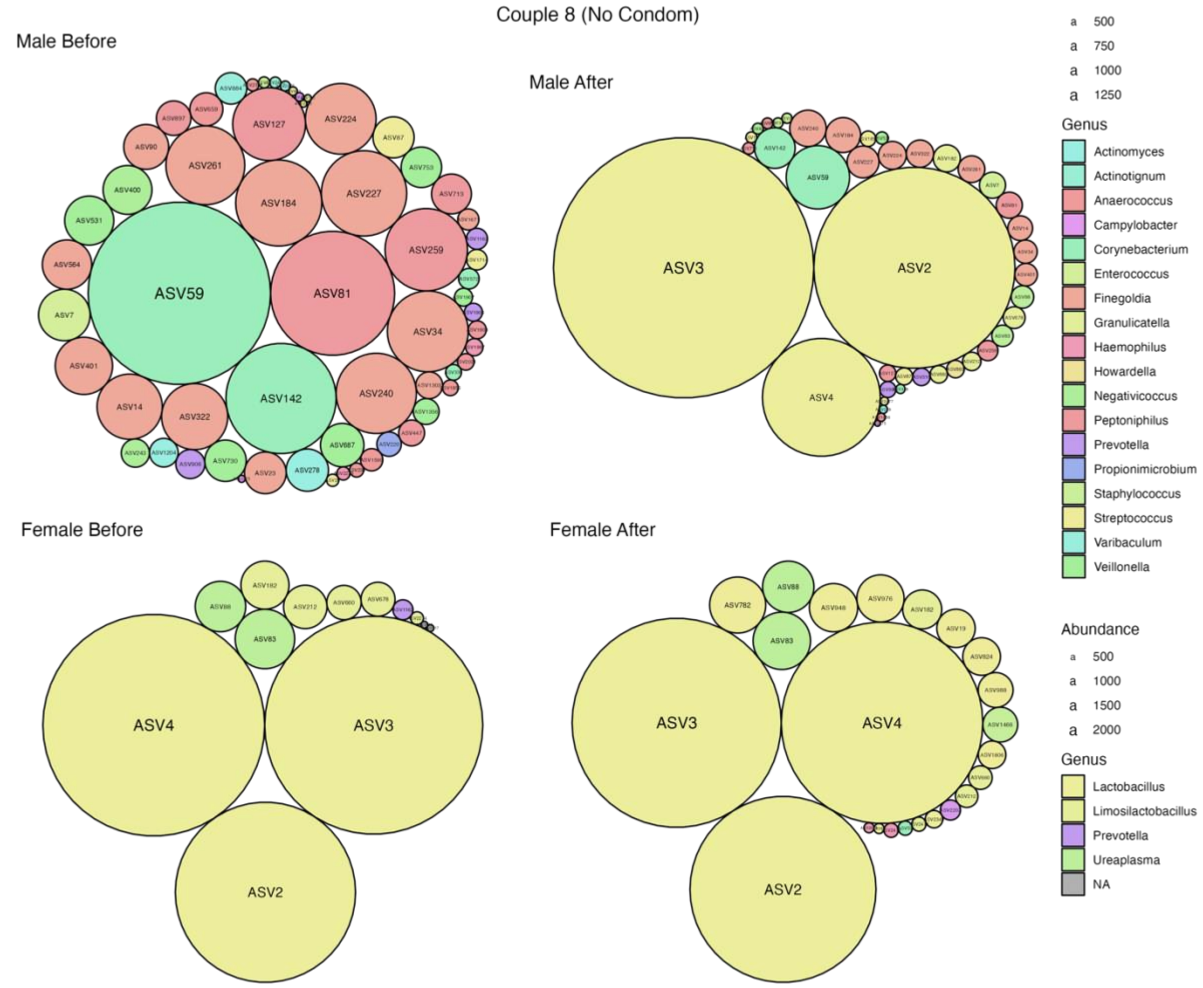

Fig S9: Taxonomic composition of the female vagina and male penile skin in couple 8. For each sample, taxonomy is visualized as circle packing. Each circle represents an ASV; relative abundance is proportional to the circle size and genus level taxonomy as colour. Samples from left to right, top to bottom: male *before*, male *after*, female *before* and female *after*.

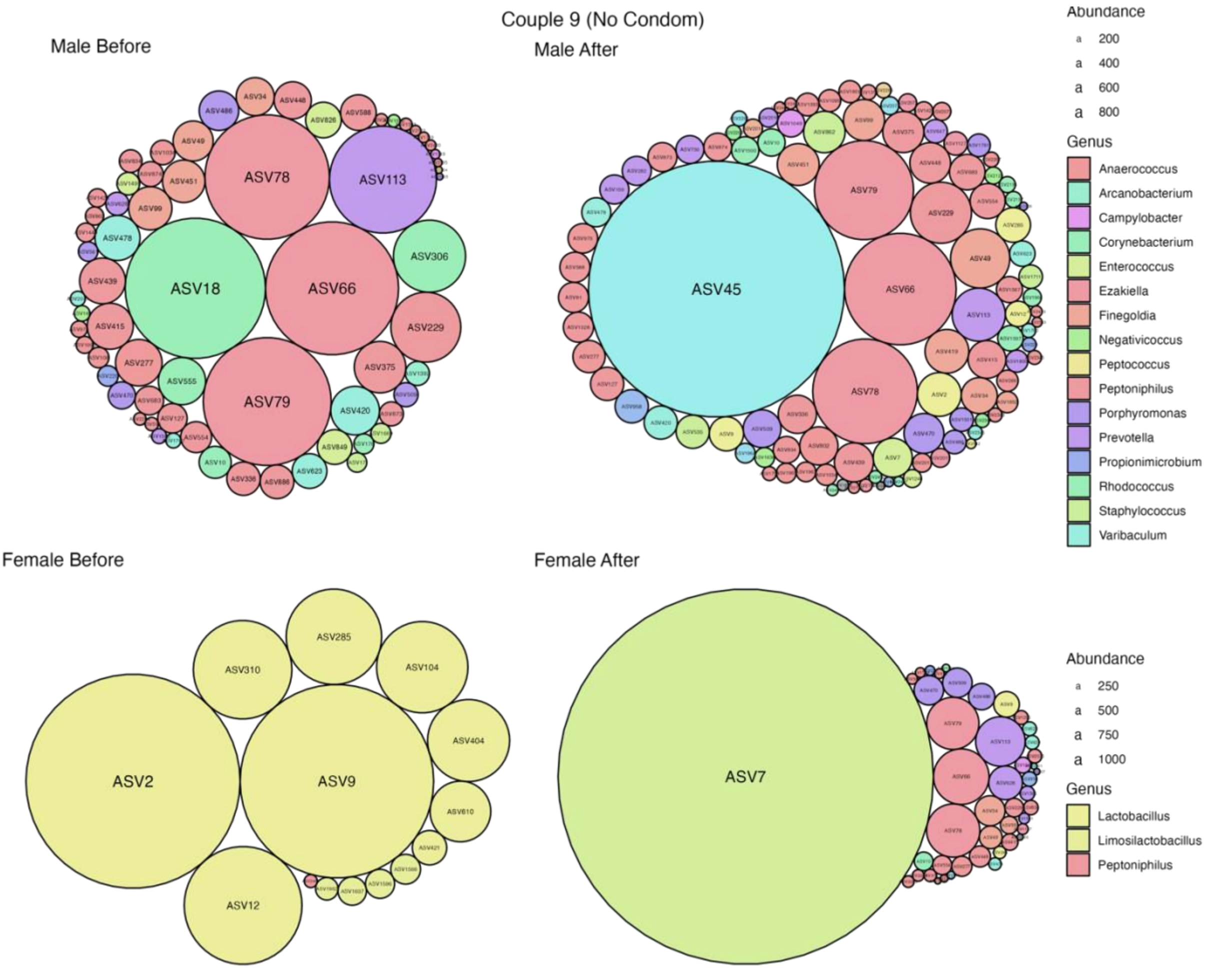

Fig S10: Taxonomic composition of the female vagina and male penile skin in couple 9. For each sample, taxonomy is visualized as circle packing. Each circle represents an ASV; relative abundance is proportional to the circle size and genus level taxonomy as colour. Samples from left to right, top to bottom: male *before*, male *after*, female *before* and female *after*.

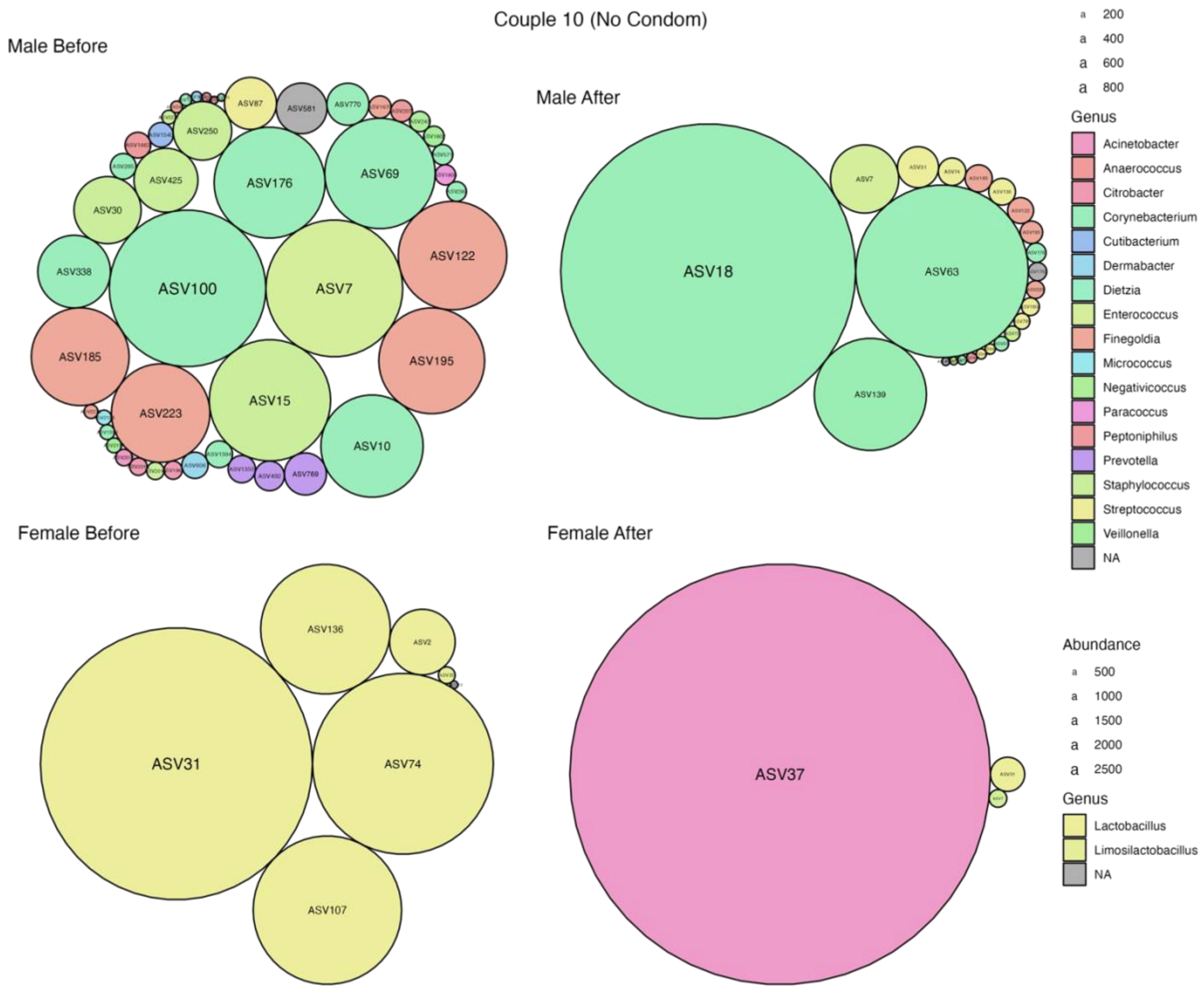

Fig S11: Taxonomic composition of the female vagina and male penile skin in couple 10. For each sample, taxonomy is visualized as circle packing. Each circle represents an ASV; relative abundance is proportional to the circle size and genus level taxonomy as colour. Samples from left to right, top to bottom: male *before*, male *after*, female *before* and female *after*.

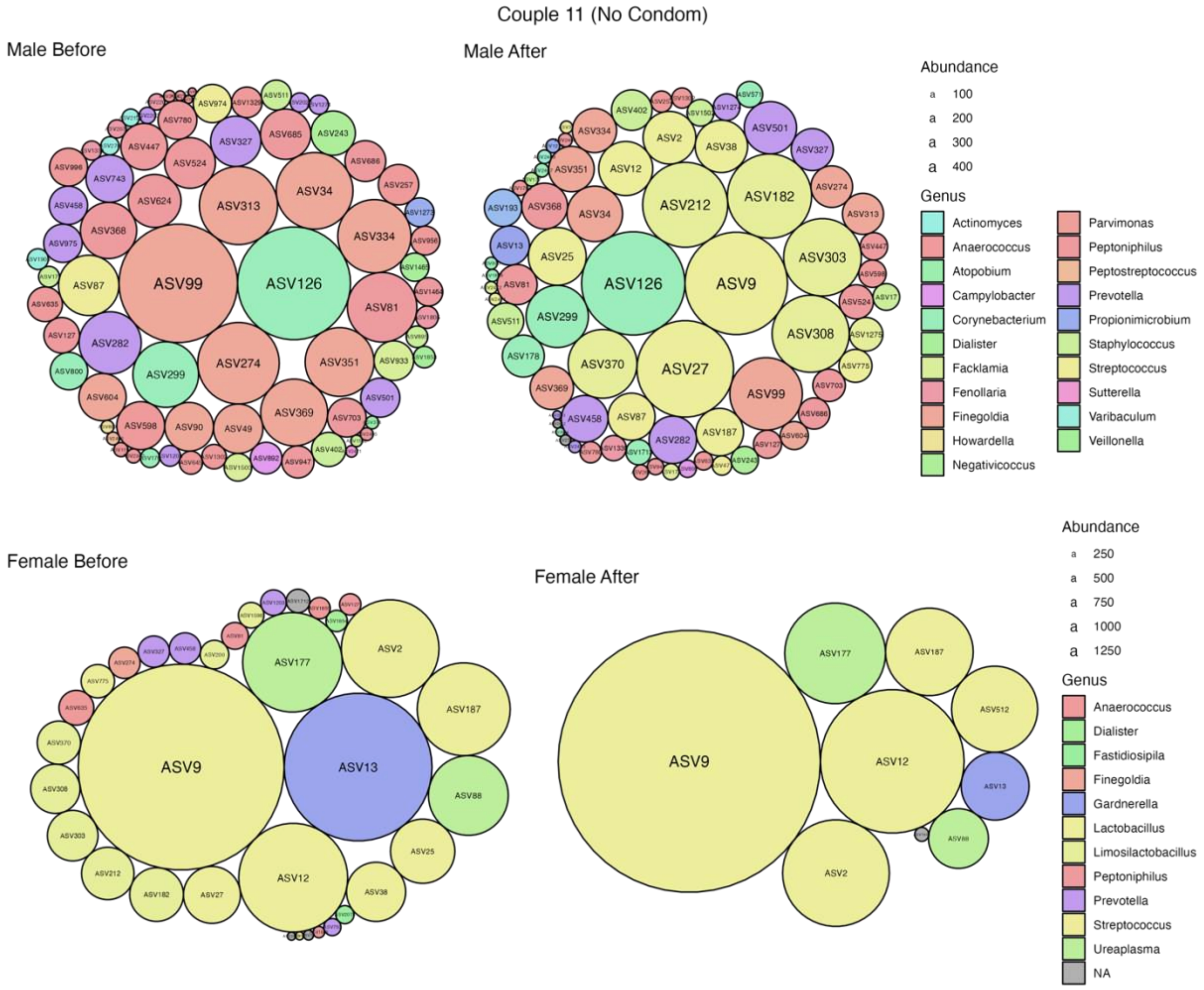

Fig S12: Taxonomic composition of the female vagina and male penile skin in couple 11. For each sample, taxonomy is visualized as circle packing. Each circle represents an ASV; relative abundance is proportional to the circle size and genus level taxonomy as colour. Samples from left to right, top to bottom: male *before*, male *after*, female *before* and female *after*.

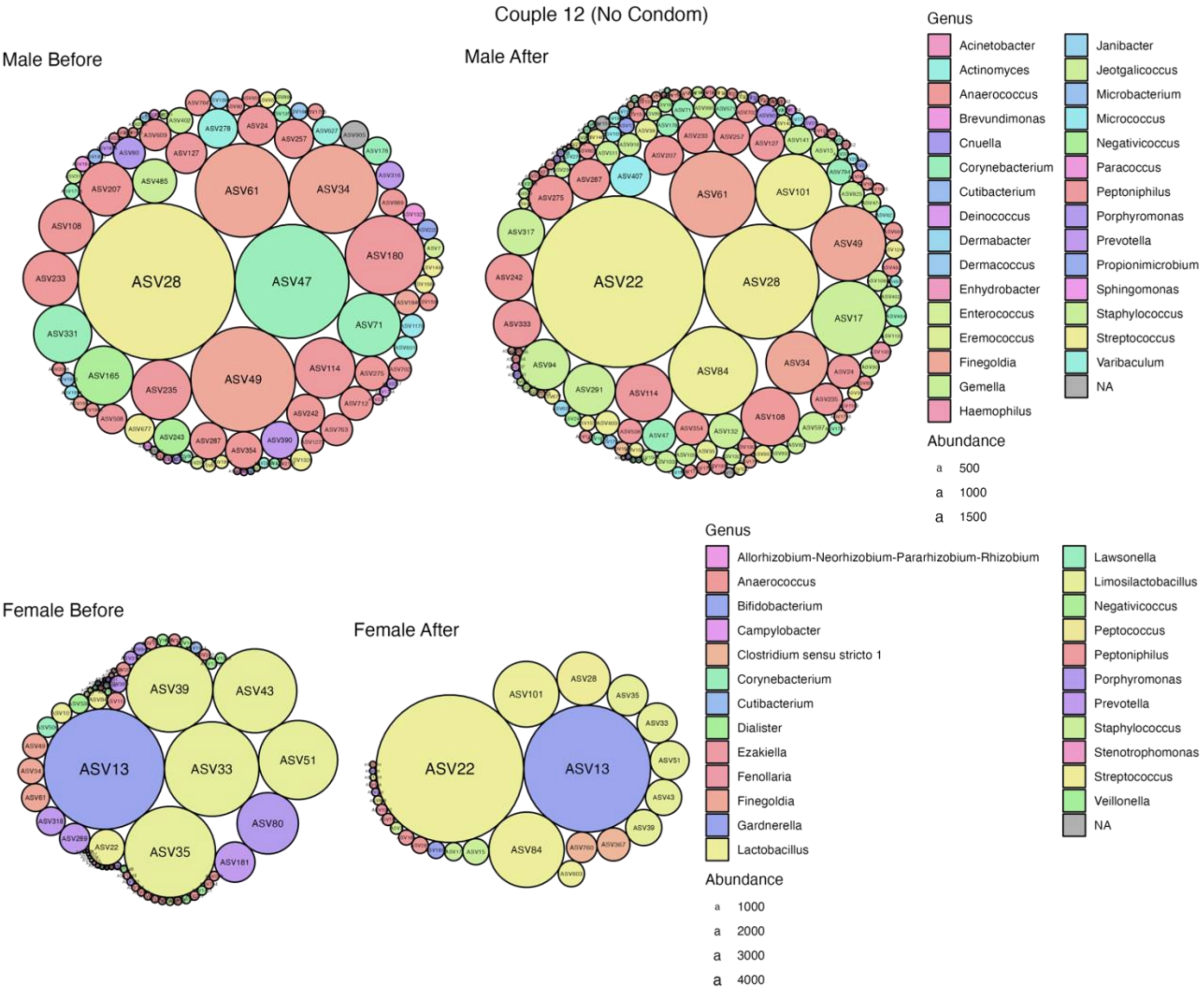

Fig S13: Taxonomic composition of the female vagina and male penile skin in couple 12. For each sample, taxonomy is visualized as circle packing. Each circle represents an ASV; relative abundance is proportional to the circle size and genus level taxonomy as colour. Samples from left to right, top to bottom: male *before*, male *after*, female *before* and female *after*.

Table S1: ZymoBIOMICS Microbial Community Standard theoretical 16S rRNA gene composition and NCBI accession numbers to reference genomes (81).

Species	Theoretical 16S Composition (%)	NCBI Reference Accession Number
Pseudomonas aeruginosa	4.2	CP117974, CP117975
Escherichia coli	10.1	CP117971, CP117972
Salmonella enterica	10.4	CP117976, CP117977 CP117978
Lactobacillus fermentum	18.4	CP132481
Enterococcus faecalis	9.9	CP117970
Staphylococcus aureus	15.5	CP117979, CP117981
Listeria monocytogenes	14.1	CP117973
Bacillus subtilis	17.4	CP118021, CP118022
Saccharomyces cerevisiae	NA	JAQZRZ000000000
Cryptococcus neoformans	NA	JAQZRY000000000

Table S2. Participant demographics

Couple	Participant Age	Medical History	Current Medications	Living with Partner/ Days per week together	Contraception	Time Abstinent Prior to Intercourse	Lubrications Used	Use of antimicrobial soaps	Oral Intercourse	Most recent underwear type	Circumsized (males only)	Public hair present	Hair Removal Method
1	Male = *	N/A	N/A	*	Contraceptive Pill	5 days	No	*	*	Cotton	No	Yes	N/A
	Female = 22	N/A	Contraceptive Pill	*				*	*	Nylon	N/A	Yes	N/A
2	Male = 25	N/A	N/A	*	Contraceptive Pill	10 days	Yes (Lifestyles Water Based)	*	*	Polyester/Cotton	Yes	Yes	N/A
	Female = 25	N/A	Minocycline, Amitriptyline, Microlut	*				*	*	Cotton	N/A	Yes	N/A
3	Male = 30	N/A	N/A	No / 5 days	Contraceptive Pill	4 days	No	No	No	Cotton	No	No	Laser
	Female = 28	N/A	Contraceptive Pill							Cotton	N/A	No	Laser
4	Male = 23	N/A	N/A	Yes	Contraceptive Pill	2 days	No	No	No	Cotton	No	Yes	*
	Female = 22	N/A	Contraceptive Pill							Cotton	N/A	Yes	N/A
5	Male = 23	N/A	Doxycycline	Yes	Contraceptive Pill	2 days	No	No	No	Nylon	No	Yes	N/A
	Female = 22	N/A	Escitalopram, Dexamphetamine, Contraceptive Pill							Cotton	N/A	No	Wax
6	Male = 22	Nut Allergy	N/A	No / 6 days	Contraceptive Pill	3 days	No	No	Yes	Polyester/Cotton	No	Yes	Razor
	Female = 22	N/A	Contraceptive Pill							Cotton	N/A	Yes	Razor
7	Male = 22	N/A	N/A	No / 7 days	Contraceptive Pill	3 days	No	No	Yes	Cotton	No	Yes	Razor
	Female = 28	N/A	Contraceptive Pill							Nylon	N/A	No	Laser
8	Male = 22	N/A	N/A	No / 7 days	Contraceptive Pill	3 days	No	No	Yes	Cotton	No	Yes	Razor
	Female = 28	N/A	Contraceptive Pill							Nylon	N/A	No	Laser
9	Male = 23	N/A	N/A	Yes	Contraceptive Pill	2 days	No	No	No	Cotton	No	Yes	*
	Female = 22	N/A	Contraceptive Pill							Cotton	N/A	Yes	N/A
10	Male = 23	N/A	Doxycycline	Yes	Contraceptive Pill	2 days	No	No	No	Nylon	No	Yes	N/A
	Female = 22	N/A	Escitalopram, Dexamphetamine, Contraceptive Pill							Cotton	N/A	No	Wax
11	Male = 22	Nut Allergy	N/A	No / 6 days	Contraceptive Pill	3 days	No	No	Yes	Polyester/Cotton	No	Yes	Razor
	Female = 22	N/A	Contraceptive Pill							Cotton	N/A	Yes	Razor
12	Male = *	N/A	N/A	No / 5 days	Contraceptive Pill	4 days	No	No	No	Cotton	No	No	Laser
	Female = 20	Ezcema	Contraceptive Pill							Cotton	N/A	No	Laser

Table S3. Table of results for alpha diversity plots between variables in the study.

Cohort	Sampling Time	Variable	Option	Alpha Diversity Measure, p-value Shannon
Male	Before	Condom Use	Yes vs No	0.1
Female				0.86
Male	After			0.018
Female				0.37
Male	Before	Cicumcision Status	Circumsized vs Uncircumsized	0.15
Female				0.37
Male	After			0.73
Female				0.86
Male	After	Lubricant Use	Yes vs No	0.86
Female				1
Male	Before	Pubic Hair*	Hair present vs No hair present	Sample size too small. No hair present, n = 1.
Female				0.82
Male	After	Oral Intercourse Occurance	Yes vs No	0.8
Female				0.8
Male	Before	Abstinence Period	2-3 days vs 4-5 days	0.63
			2-3 days vs 6-10 days	1
			4-5 days vs 6-10 days	1
Female			2-3 days vs 4-5 days	0.86
			2-3 days vs 6-10 days	0.63
			4-5 days vs 6-10 days	1

* Survey asked participants how much pubic hair is present. The options were natural, minimally trimmed, mostly trimmed and no hair. For the purpose of this analysis these have been recategorised as hair present or no hair present.

Table S4. Contaminants identified from the *decontam* package and removed from the data.

ASV ID	Phylum	Genus	Species
ASV97	Firmicutes	Parvimonas	NA
ASV1382	Firmicutes	Finegoldia	NA
ASV700	Firmicutes	Finegoldia	NA
ASV472	Firmicutes	Peptostreptococcus	anaerobius
ASV684	Firmicutes	S5-A14a	NA
ASV216	Firmicutes	Agathobacter	NA
ASV148	Firmicutes	Mycoplasma	NA
ASV232	Firmicutes	Mycoplasma	NA
ASV847	Firmicutes	Mycoplasma	NA
ASV268	Firmicutes	Mycoplasma	NA
ASV135	Firmicutes	Holdemanella	NA
ASV1051	Firmicutes	Phascolarctobacterium	NA
ASV563	Firmicutes	NA	NA
ASV245	Firmicutes	Dialister	NA
ASV896	Firmicutes	Dialister	NA
ASV567	Firmicutes	Veillonella	NA
ASV1071	Firmicutes	UCG-002	NA
ASV192	Firmicutes	Ruminococcus	NA
ASV442	Firmicutes	Subdoligranulum	NA
ASV1228	Firmicutes	Fournierella	NA
ASV1100	Firmicutes	Christensenellaceae R-7 group	NA
ASV618	Firmicutes	Christensenellaceae R-7 group	NA
ASV175	Actinobacteriota	Lawsonella	NA
ASV40	Actinobacteriota	Cutibacterium	acnes
ASV661	Actinobacteriota	Bifidobacterium	animalis
ASV158	Bacteroidota	Porphyromonas	NA
ASV845	Bacteroidota	Bacteroides	NA
ASV811	Bacteroidota	Bacteroides	caccae
ASV652	Bacteroidota	Bacteroides	uniformis
ASV614	Bacteroidota	Prevotella	NA
ASV583	Bacteroidota	Prevotella_9	NA
ASV536	Bacteroidota	Prevotella	bivia
ASV525	Bacteroidota	Parabacteroides	NA
ASV1159	Campylobacterota	Campylobacter	NA
ASV6	Proteobacteria	Burkholderia-Caballeronia-Paraburkholderia	NA
ASV11	Proteobacteria	Burkholderia-Caballeronia-Paraburkholderia	NA
ASV16	Proteobacteria	Burkholderia-Caballeronia-Paraburkholderia	NA
ASV204	Proteobacteria	Escherichia-Shigella	NA
ASV258	Proteobacteria	Escherichia-Shigella	NA
ASV284	Proteobacteria	Escherichia-Shigella	NA
ASV436	Proteobacteria	Escherichia-Shigella	NA
ASV872	Firmicutes	Peptoniphilus	NA
ASV987	Firmicutes	Peptoniphilus	NA
ASV587	Firmicutes	Peptoniphilus	NA
ASV795	Firmicutes	Peptoniphilus	NA
